# Functional and clinical significance of the RNA m^6^A methyltransferase complex in breast cancer

**DOI:** 10.1038/s41523-025-00861-5

**Published:** 2025-11-27

**Authors:** Anna E. Harris, Jennifer Lothion-Roy, Rachel L. Thompson, Maria Haque, Corinne L. Woodcock, Mansour A. Alsaleem, Alexander Dean, Yousif Kariri, Michael S. Toss, Lorraine J. Gudas, Andrew R. Green, Cinzia Allegrucci, Melissa B. Davis, Sheeba Irshad, Kyong-Hwa Park, Srinivasan Madhusudan, Rupert G. Fray, Jennie N. Jeyapalan, Catrin S. Rutland, Emad A. Rakha, Nigel P. Mongan

**Affiliations:** 1 Biodiscovery Institute, University of Nottingham, Nottingham, UK; 2 School of Veterinary Medicine and Science, University of Nottingham, Nottingham, UK; 3 Unit of Scientific Research, Applied College, Qassim University, Qassim, Saudi Arabia; 4 Nottingham Breast Cancer Research Centre, School of Medicine, University of Nottingham, Nottingham, UK; 5 Department of Clinical Laboratory Science, College of Applied Medical Science, Shaqra University, Shaqra, Saudi Arabia; 6 Histopathology Department, Sheffield Teaching Hospitals NHS Foundation Trust, Sheffield, UK; 7 Department of Pharmacology, Weill Cornell Medicine, New York, NY USA; 8 Department of Microbiology, Biochemistry and Immunology, and Institute of Translational Genomic Medicine, Morehouse School of Medicine, Atlanta, GA USA; 9 School of Cancer & Pharmaceutical Sciences, King’s College London & Guys & St Thomas NHS Trust, London, UK; 10 College of Medicine, Korea University, Korea, Republic of Korea; 11 School of Biosciences, University of Nottingham, Nottingham, UK; 12 Nottingham University NHS Trust, Nottingham, UK

**Keywords:** Cancer, Cell biology, Molecular biology, Biomarkers, Medical research, Oncology, Pathogenesis

## Abstract

The RNA modification N^6^-methyladenosine (m^6^A) plays a key role in RNA processing. It is catalysed by the RNA methyltransferase complex (MTC) which includes METTL3, METTL14 and CBLL1. Recently, a METTL3 inhibitor demonstrated promising preclinical results in several cancer types, yet the therapeutic potential of targeting m^6^A in breast cancer (BCa) remains poorly understood. Utilising a large BCa cohort, we identified that increased METTL14 and CBLL1 expression was associated with a more favourable prognosis, whereas increased METTL3 expression was associated with poorer patient outcomes in Triple Negative BCa (TNBC). Using siRNA depletion, we identified distinct METTL3, METTL14 and CBLL1 regulated gene networks in BCa cell lines. METTL3 inhibition reduced proliferation and invasion of BCa cell lines and induced an immune activation transcriptional signature. These results provide insight into the clinical functions of METTL3, METTL14 and CBLL1 in BCa and support the therapeutic potential of targeting METTL3 in BCa, particularly in TNBC.

## Introduction

Breast cancer (BCa) is the most commonly diagnosed malignancy and the leading cause of cancer-related deaths in women globally^1,2^. BCa tumours are sub-grouped based on their hormone receptor status, human epidermal growth factor receptor 2 (HER2) status and intrinsic molecular subtypes^3^. Hormone receptor (estrogen receptor (ER) and progesterone receptor (PR))-positive luminal tumours, accounting for 70-80% of all BCa, are treated with hormone therapy but still a proportion recurs^4,5^. Triple negative (ER, PR and HER2 negative) BCa (TNBC), which is diagnosed in ~10% of BCa patients, is associated with a poor prognosis due to an aggressive phenotype and a lack of specific therapeutic targets^3,6–9^. Therefore, there is an urgent need for novel therapeutic approaches for BCa.

The methylation of the N^6^ position of adenosine (m^6^A) is the most prevalent RNA modification in eukaryotic cells^10^. Enzymatic writers and erasers of m^6^A catalyse the dynamic and reversible addition and removal of the methyl group, whilst readers bind to influence RNA processing, including mRNA splicing, translation, localisation and stability^11,12^. The m^6^A methyltransferase complex (MTC) (writer complex) comprises the catalytic subunit METTL3, bound in a stable complex with proteins including METTL14, CBLL1 (HAKAI), WTAP, VIRMA (KIAA1429), ZC3H13, RBM15, and its paralogue RBM15B^13–17^.

The MTC components have been reported to have complex roles in BCa and the relevance of the RNA MTC and m^6^A as therapeutic targets in BCa remains poorly understood. Previous studies examining the functions of METTL3 and METTL14 in BCa have reported both tumour suppressor and oncogenic roles and have implicated METTL3 and METTL14 in several mechanisms^18–24^. CBLL1 is an adaptor protein that binds to the METTL3-METTL14 heterodimer and is believed to be required for full m^6^A methylation activity^17,25^. Conflicting findings have also been reported in studies investigating CBLL1 in BCa. CBLL1 has been positively associated with increased invasive potential and metastasis of BCa cells^26,27^, whilst other authors reported an association between higher CBLL1 expression and better relapse-free and overall survival (OS) in BCa patients^28^.

Recently, a first-in-class METTL3 inhibitor has been reported^29^. This small molecule inhibitor, STM2457, is highly selective for the S-adenosyl methionine (SAM) binding site of METTL3 and was found to reduce m^6^A levels and have promising effects in several cancer contexts^30–33^. STC-15, a potent derivative of STM2457, has entered Phase I clinical trial for solid tumours (NCT05584111). METTL3 inhibition has been shown to induce an anti-cancer immune response and it has been suggested, therefore, that METTL3 inhibitors may complement immunomodulatory therapies, including anti-PD1 therapy^34^.

In this study, we evaluated the expression and clinical relevance of METTL3, METTL14 and CBLL1 in large publicly available BCa datasets (RNA level) and in a well-characterised operable BCa patient cohort (~2500 patients) by immunohistochemical (IHC) staining. To provide insights into the functional effects of RNA methylation in BCa, we examined the effects of siRNA knockdown of METTL3, METTL14 and CBLL1 on the transcriptome of BCa cell lines by RNA-sequencing (RNA-seq). Finally, the effects of the STM2457 METTL3 inhibitor on BCa cell phenotype and transcriptome were assessed.

## Results

### METTL3, METTL14 and CBLL1 expression in breast cancer

**Fig. 1:**
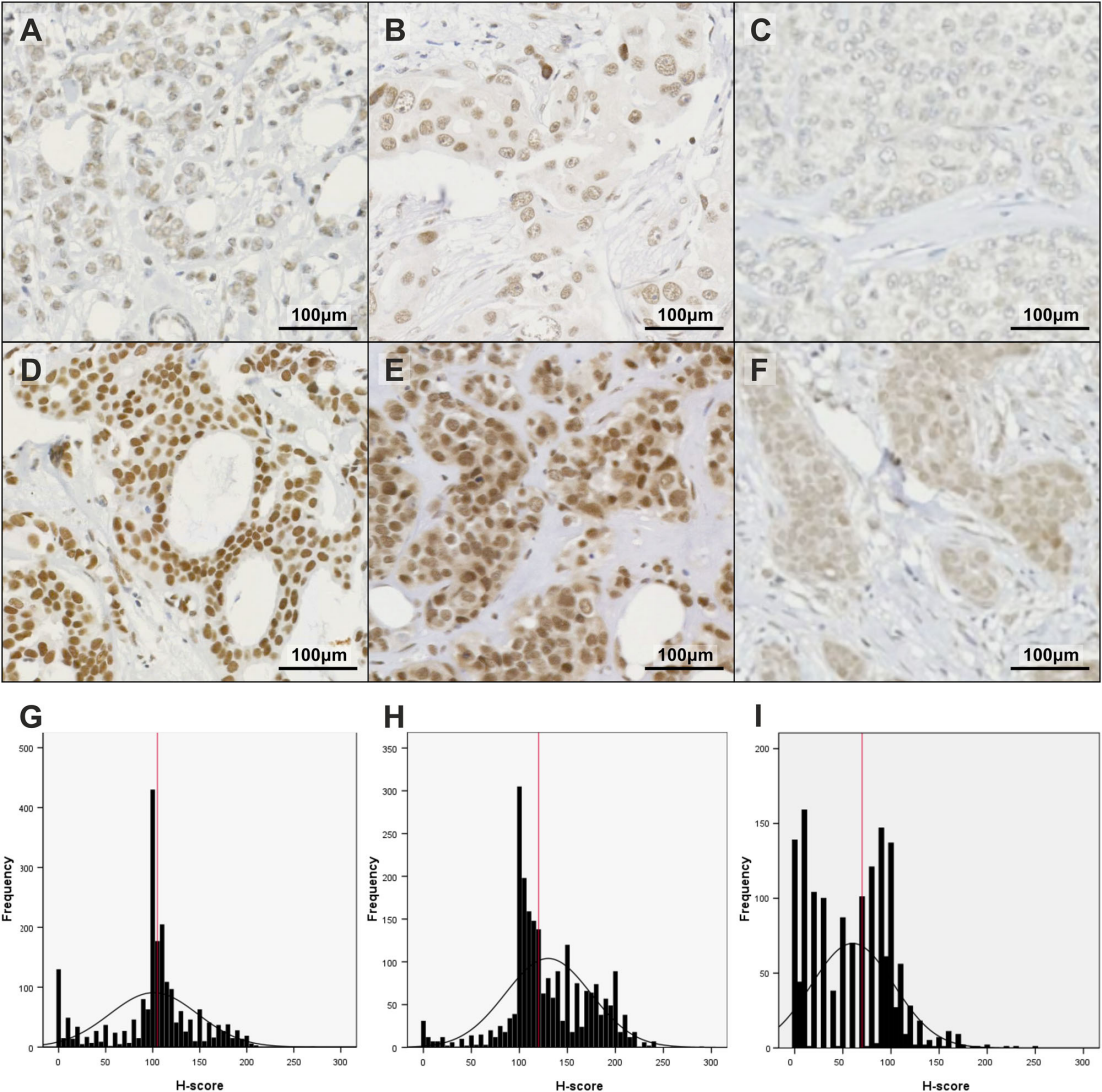
Representative images of IHC nuclear staining of METTL3, METTL14 and CBLL1 and distribution of H-scores in breast cancer tissue microarrays (TMAs). Representative photomicrographs of (**A**) low and (**D**) high level METTL3 expression; (**B**) low and (**E**) high level METTL14 expression; and (**C**) low and (**F**) high level CBLL1 expression. H-scores assigned to intact cores with tumour coverage >10%. Distribution of H-scores with red line denoting median: (**G**) METTL3, median H-score 105 (n = 2132); (**H**) METTL14, median H-score 120 (n = 2321), and (**I**) CBLL1, median H-score 70 (n = 1524).

Within the TCGA Breast Invasive Carcinoma PanCancer Atlas dataset (n = 996) mutations of *METTL3*, *METTL14* and *CBLL1* were uncommon, with amplifications being the most common genetic alteration (Supplementary Fig. 1A). Comparison of tumour and normal mRNA expression using TCGA GDC data (n = 1284) revealed no significant difference for *METTL3* or *CBLL1* expression, whereas *METTL14* expression was lower in tumour tissue compared with normal breast tissue (p < 0.0001) (Supplementary Fig. 1B-D). KM plots revealed increased *METTL14* and *CBLL1* expression correlated with increased OS whereas *METTL3* showed no significant correlation (Supplementary Fig. 1 E-G). Regarding protein expression, varying intensities of METTL3, METTL14 and CBLL1 staining was observed in the nuclei of tumour epithelial cells by IHC (Fig. 1). H-scores obtained for METTL3 (n = 2132), METTL14 (n = 2321) and CBLL1 (n = 1524) were correlated with clinicopathological data.

### Clinicopathological correlations associated with METTL3, METTL14 and CBLL1 expression

High METTL3 expression was significantly associated with lower grade, lower pleomorphic score, low mitotic score, ER and PR positivity and non-TNBC status (all p < 0.05; Table 1). Correlations with molecular class revealed high nuclear METTL3 expression to be associated with luminal A and luminal B, whilst lower METTL3 expression was associated with TNBC and HER2 enriched molecular subtypes (Table 1).

**Table 1:** Correlation of patient demographics and clinicopathological parameters with IHC nuclear staining of METTL3, METTL14 and CBLL1 in breast cancer TMAs

Clinicopathological criteria	METTL3 expression (%)		χ² (p value)	METTL14 expression (%)		χ² (p value)	CBLL1 expression (%)		χ² (p value)
	Low (≤105)	High (≥106)		Low (≤120)	High (≥121)		Low (≤70)	High (≥71)	
**Tumour grade**									
1	180 (53.6%)	156 (46.4%)	**8.221 (0.016)**	109 (32.2%)	229 (68.7%)	**136.645 (** < **0.0001)**	81 (42.6%)	109 (57.4%)	**77.782 (** < **0.0001)**
2	448 (54.8%)	370 (45.2%)		380 (45.4%)	457 (54.6%)		228 (44.7%)	282 (55.3%)	
3	582 (60.6%)	379 (39.4%)		723 (64.4%)	399 (35.6%)		542 (66.7%)	271 (33.3%)	
**Tubular differentiation**									
T1	80 (51.0%)	77 (49.0%)	5.115 (0.078)	45 (33.1%)	91 (66.9%)	**61.594 (** < **0.0001)**	36 (43.9%)	46 (56.1%)	**25.987 (** < **0.0001)**
T2	358 (55.2%)	290 (44.8%)		324 (44.6%)	403 (55.4%)		220 (48.2%)	236 (51.8%)	
T3	772 (58.9%)	538 (41.1%)		843 (58.8%)	591 (41.2%)		595 (61.0%)	380 (39.0%)	
**Nuclear pleiomorphism**									
P1	25 (50.0%)	25 (50.0%)	**7.624 (0.022)**	17 (33.3%)	34 (66.7%)	**97.764 (** < **0.0001)**	18 (54.5%)	15 (45.5%)	**20.805 (** < **0.0001)**
P2	379 (53.5%)	329 (46.5%)		272 (38.5%)	435 (61.5%)		202 (47.1%)	227 (52.9%)	
P3	806 (59.4%)	551 (40.6%)		923 (60.0%)	616 (40.0%)		631 (60.0%)	420 (40.0%)	
**Mitotic count**									
M1	520 (56.0%)	408 (44.0%)	**15.737 (** < **0.0001)**	377 (40.6%)	551 (59.4%)	**105.342 (** < **0.0001)**	210 (39.8%)	318 (60.2%)	**114.664 (** < **0.0001)**
M2	219 (50.8%)	212 (49.2%)		247 (54.0%)	210 (46.0%)		158 (53.0%)	140 (47.0%)	
M3	471 (62.3%)	285 (37.7%)		588 (64.5%)	324 (35.5%)		483 (70.3%)	204 (29.7%)	
**Lymph node status**									
Negative	738 (57.0%)	557 (43.0%)	0.056 (0.814)	711 (51.0%)	684 (49.0%)	5.395 (0.067)	516 (56.7%)	394 (43.3%)	0.194 (0.659)
Positive	471 (57.5%)	348 (42.5%)		500 (55.5%)	401 (44.5%)		335 (55.6%)	268 (44.4%)	
**Lymph node stage**									
1	738 (57.0%)	557 (43.0%)	3.077 (0.215)	711 (51.0%)	684 (49.0%)	4.502 (0.105)	516 (56.7%)	389 (43.3%)	0.272 (0.878)
2	346 (55.8%)	274 (44.2%)		376 (55.5%)	301 (44.5%)		257 (55.9%)	203 (44.1%)	
3	125 (62.8%)	74 (37.2%)		124 (55.4%)	100 (44.6%)		78 (54.5%)	65 (45.5%)	
**Tumour size**									
<2 cm	687 (56.6%)	526 (43.4%)	0.383 (0.536)	611 (48.1%)	659 (51.9%)	**24.688 (** < **0.0001)**	408 (51.6%)	383 (48.4%)	**14.662 (** < **0.0001)**
≥2 cm	523 (58.0%)	379 (42.0%)		601 (58.5%)	426 (41.5%)		443 (61.4%)	279 (38.6%)	
**Nottingham Prognostic Index Category**									
Good prognostic group	385 (55.1%)	314 (44.9%)	4.089 (0.129)	286 (39.8%)	432 (60.2%)	**76.447 (** < **0.0001)**	194 (45.5%)	232 (54.5%)	**28.872 (** < **0.0001)**
Moderate prognostic group	619 (57.2%)	464 (42.8%)		676 (56.9%)	512 (43.1%)		473 (59.4%)	323 (40.6%)	
Poor prognostic group	205 (61.7%)	127 (38.3%)		249 (63.8%)	141 (36.2%)		184 (63.2%)	107 (36.8%)	
**Histological tumour type**									
NST	779 (59.6%)	529 (40.4%)	**9.885 (0.042)**	883 (59.3%)	605 (40.7%)	**85.061 (** < **0.0001)**	633 (60.7%)	410 (39.3%)	**35.049 (** < **0.0001)**
ILC	118 (57.3%)	88 (42.7%)		78 (41.3%)	111 (58.7%)		42 (41.6%)	59 (58.4%)	
Metaplastic carcinoma	1 (50.0%)	1 (50.0%)		8 (80.0%)	2 (20.0%)		6 (100.0%)	0 (0.0%)	
Pure special tumour type	51 (49.0%)	53 (51.0%)		24 (28.2%)	61 (71.8%)		25 (59.0%)	26 (51.0%)	
Mixed NST & other type	261 (52.7%)	234 (47.3%)		219 (41.7%)	306 (58.3%)		145 (46.5%)	167 (53.5%)	
**Molecular class**									
Luminal A	385 (54.8%)	318 (45.2%)	**21.913 (** < **0.0001)**	280 (38.1%)	454 (61.9%)	**145.519 (** < **0.0001)**	159 (38.4%)	255 (61.6%)	**90.114 (** < **0.0001)**
HER2 enriched	70 (72.9%)	26 (27.1%)		90 (71.4%)	36 (28.6%)		67 (68.4%)	31 (31.6%)	
Triple negative	215 (65.3%)	114 (34.7%)		287 (72.7%)	108 (27.3%)		214 (70.9%)	88 (29.1%)	
Luminal B	352 (54.7%)	292 (45.3%)		396 (54.8%)	327 (45.2%)		301 (60.9%)	193 (39.1%)	
**Vascular invasion**									
Absent	843 (57.7%)	619 (42.3%)	0.392 (0.531)	795 (50.9%)	768 (49.1%)	**7.090 (0.008)**	552 (55.3%)	446 (44.7%)	1.042 (0.307)
Present	367 (56.2%)	286 (43.8%)		417 (56.8%)	317 (43.2%)		299 (58.1%)	216 (41.9%)	
**Ki67 index group**									
Low	427 (54.6%)	355 (45.4%)	2.569 (0.109)	338 (40.7%)	493 (59.3%)	**59.146 (** < **0.0001)**	205 (42.4%)	279 (57.6%)	**55.679 (** < **0.0001)**
High	478 (58.6%)	338 (41.4%)		561 (58.9%)	391 (41.1%)		450 (64.3%)	250 (35.7%)	
**ER status**									
Negative	311 (66.3%)	158 (33.7%)	**20.292 (** < **0.0001)**	399 (71.3%)	161 (28.7%)	**101.073 (** < **0.0001)**	301 (70.3%)	127 (29.7%)	**48.436 (** < **0.0001)**
Positive	895 (54.6%)	743 (45.4%)		810 (46.8%)	919 (53.2%)		546 (50.6%)	533 (49.4%)	
**PR status**									
Negative	529 (62.2%)	322 (37.8%)	**12.827 (** < **0.0001)**	606 (63.0%)	356 (37.0%)	**68.835 (** < **0.0001)**	435 (65.2%)	232 (34.8%)	**38.865 (** < **0.0001)**
Positive	658 (54.2%)	555 (45.8%)		587 (45.4%)	707 (54.6%)		401 (49.1%)	416 (50.9%)	
**HER2 status**									
Negative	1027 (56.7%)	784 (43.3%)	3.329 (0.068)	990 (50.8%)	957 (49.2%)	**26.497 (** < **0.0001)**	681 (54.3%)	574 (45.7%)	**12.796 (** < **0.0001)**
Positive	166 (62.6%)	99 (37.4%)		201 (66.8%)	102 (33.7%)		151 (67.1%)	74 (32.9%)	
**Triple negative status**									
Non-TN	965 (55.7%)	769 (44.3%)	**10.623 (0.001)**	900 (48.5%)	955 (51.5%)	**76.145 (** < **0.0001)**	613 (52.1%)	564 (47.9%)	**34.384 (** < **0.0001)**
TN	215 (65.3%)	114 (34.7%)		287 (72.7%)	108 (27.3%)		214 (70.9%)	88 (29.1%)	
**Age**									
≤49 years	388 (57.1%)	292 (42.9%)	0.009 (0.923)	445 (57.6%)	328 (42.4%)	**10.786 (** < **0.0001)**	315 (57.6%)	232 (42.4%)	0.626 (0.429)
≥50 years	822 (57.3%)	613 (42.7%)		767 (50.3%)	757 (49.7%)		536 (55.5%)	430 (44.5%)	
**Menopausal status**									
Pre	436 (56.6%)	334 (43.4%)	0.170 (0.680)	485 (55.7%)	385 (44.3%)	**4.999 (0.025)**	339 (55.4%)	273 (44.6%)	0.304 (0.581)
Post	774 (58.0%)	571 (42.5%)		727 (50.9%)	700 (49.1%)		512 (56.8%)	389 (43.2%)	

The number (and percentage) of patients in each parameter group, with statistically significant correlations highlighted in bold. Significance determined by χ².

*NST* invasive breast cancer of no special type, *ILC* invasive lobular carcinoma, *ER* estrogen receptor, *PR* progesterone receptor, *HER2* human epidermal growth factor receptor 2, *TN* triple negative breast cancer.

Higher nuclear METTL14 expression was associated with smaller tumour size (<2cm), older age (>50 years), lower grade, with lower pleomorphism, high tubule formation and lower mitosis scores and a more favourable Nottingham Prognostic Index (NPI) group (p < 0.0001; Table 1). Higher expression was associated with non-TNBC status, with ER and PR positivity and HER2 negativity, presence of ductal carcinoma in situ (DCIS) and lobular carcinoma in situ (LCIS), lack of lymphovascular invasion (LVI) and lower Ki67 proliferation index. Lower METTL14 expression was associated with TNBC and HER2 enriched molecular subtypes (p < 0.0001; Table 1).

Nuclear CBLL1 expression was significantly higher in patients with a lower grade at diagnosis, a favourable NPI prognostic group, ER positive status, and with non-TNBC status (p < 0.0001; Table 1). Similarly, higher nuclear CBLL1 was significantly associated with a smaller tumour size, lower Ki67 labelling index, PR positivity and HER2 negativity, as well as histological tumour types of a more favourable prognosis (p < 0.0001; Table 1).

### METTL3 expression negatively associated and CBLL1 and METTL14 expression positively associated with increased patient survival

**Fig. 2:**
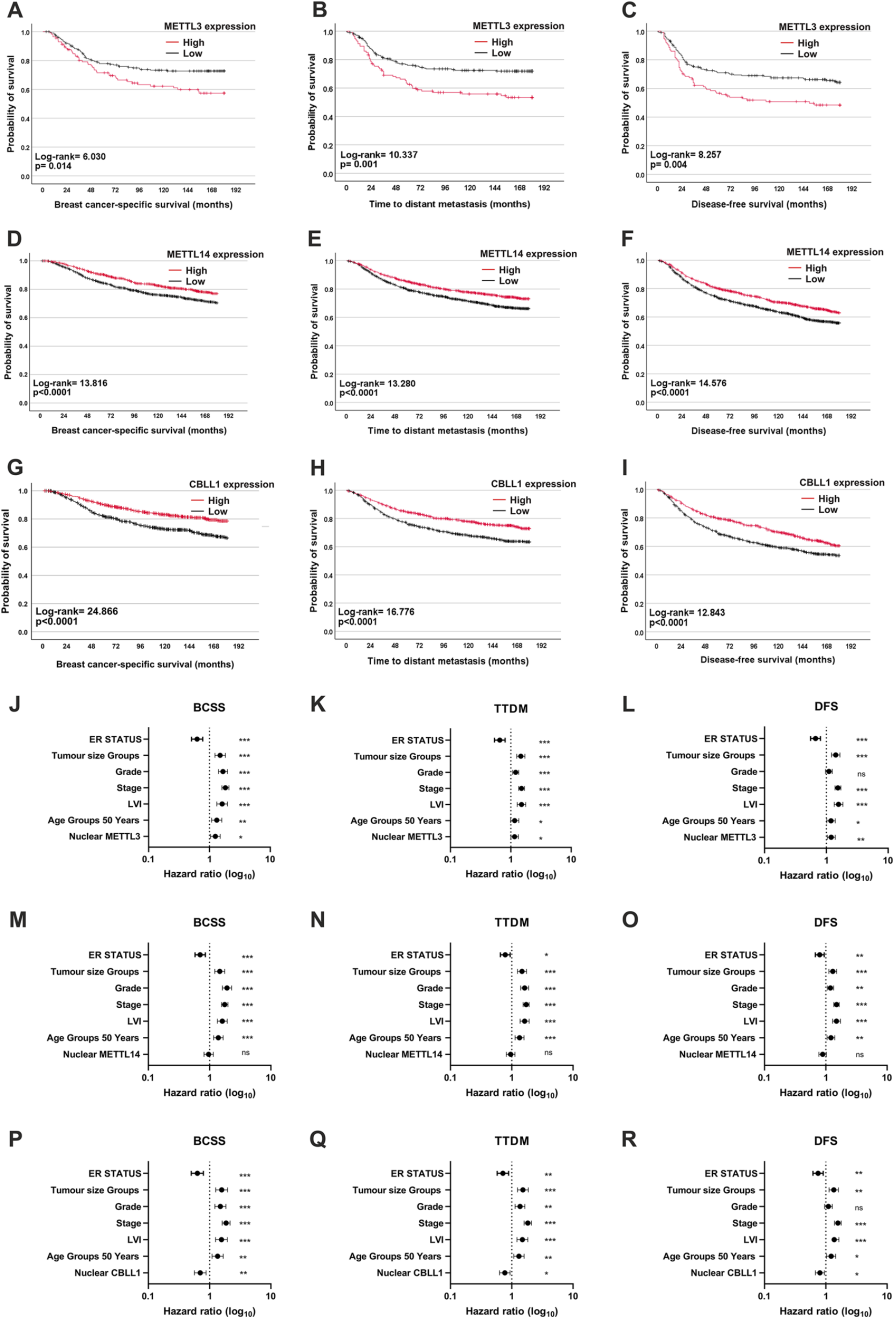
METTL3, METTL14 and CBLL1 expression correlated with survival and prognosis in breast cancer patients. IHC staining of BCa patient samples was assessed by H-score, divided by median into high and low expression groups and correlated with breast cancer-specific survival (BCSS), time to distant metastasis (TTDM) and disease-free survival (DFS). METTL3 staining in TNBC patients only, low: ≤105, high: ≥106. METTL14 staining in all BCa patients, low: ≤120, high: ≥121. CBLL1 staining in all BCa patients, low: ≤70, high: ≥71. Correlations: (**A**) METTL3 and BCSS in TNBC (n = 329, events at 15 years, low: 56/215, high: 44/114), (**B**) METTL3 and TTDM in TNBC (n = 329, events at 15 years, low: 58/215, high: 51/114), (**C**) METTL3 and DFS in TNBC (n = 329, events at 15 years, low: 73/215, high: 57/114), (**D**) METTL14 and BCSS (n = 2297, events at 15 years, low: 324/1212, high: 223/1085), (**E**) METTL14 and TTDM (n = 2297, events at 15 years, low: 379/1212, high: 269/1085), (**F**) METTL14 and DFS (n = 2297, events at 15 years, low: 494/1212, high: 366/1085). (**G)** CBLL1 and BCSS (n = 1513, events at 15 years, low: 263/851, high: 127/662), (**H**) CBLL1 and TTDM (n = 1513, events at 15 years, low: 294/851, high: 164/662), (**I**) CBLL1 and DFS (n = 1513, events at 15 years low: 374/851, high: 235/662). Correlated using Kaplan Meier estimate and analysed by log-rank test. Combined multivariate Cox regression analysis of METTL3 nuclear expression, tumour stage, tumour size, tumour grade, patient age, ER status and LVI with 15-year (**J**) BCSS, (**K**) TTDM and (**L**) DFS (n = 2106). Combined multivariate Cox regression analysis of METTL14 nuclear expression, tumour stage, tumour size, patient age, ER status and LVI with 15-year (**M**) BCSS, (**N**) TTDM and (**O**) DFS (n = 2296). Combined multivariate Cox regression analysis of CBLL1 nuclear expression, tumour stage, tumour size, tumour grade, patient age, ER status and LVI with 15-year (**P**) BCSS, (**Q**) TTDM and (**R**) DFS (n = 1507). * = p ≤ 0.05, ** = p ≤ 0.01, *** = p ≤ 0.001, ns = not significant.

To determine associations between METTL3, METTL14 and CBLL1 expression and survival outcomes, Kaplan-Meier estimates were performed. Considering the cohort as a whole, there was no significant association between METTL3 and outcomes for breast cancer-specific survival (BCSS), time to distant metastasis (TTDM) and disease-free survival (DFS) (Supplementary Fig. 2). However, in TNBC patients, high METTL3 expression was associated with decreased 15-year BCSS, TTDM and DFS (Fig. 2A–C). Higher nuclear METTL14 and higher nuclear CBLL1 expression were both significantly associated with longer BCSS, DFS and TTDM across the entire patient cohort (p < 0.001; Fig. 2D–I). This was consistent with transcriptomic data which showed a significant association between higher *CBLL1* and *METTL14* expression and increased OS (n = 2976) (Supplementary Fig. 1).

Multivariate Cox regression analyses were conducted to assess the independent prognostic value of METTL3, METTL14 and CBLL1 expression whilst accounting for the potential confounding variables of nodal stage, tumour size, patient age, ER status and LVI (Fig. 2). High nuclear METTL3 expression was significantly associated with shorter BCSS, TTDM and DFS independent of patient age, ER status, LVI, nodal stage, grade and size in both the whole cohort (P < 0.05, Fig. 2J–L) and in the TNBC subgroup of patients (P < 0.05, Supplementary Fig. 2). In contrast, nuclear CBLL1 expression was an independent positive prognostic factor for BCSS, TTDM and DFS (P < 0.05, Fig. 2P–R). Nuclear METTL14 expression was not an independent prognostic factor (Fig. 2M–O).

### Correlations between METTL3, METTL14 and CBLL1 expression and other functionally relevant proteins

Expression of METTL3, METTL14 and CBLL1 was subsequently correlated with expression of a number of other functionally related proteins previously analysed by IHC in the Nottingham BCa TMA. A positive correlation was observed between METTL3, METTL14 and CBLL1. Nuclear METTL3, METTL14 and CBLL1 were all significantly positively correlated with ERα and ER-regulated genes including AR, and FOXA1, E-cadherin, C-MYC and BRCA1 expression (p < 0.0001; Supplementary Table 1).

### METTL3, METTL14 and CBLL1 regulate distinct transcriptional networks

**Fig. 3:**
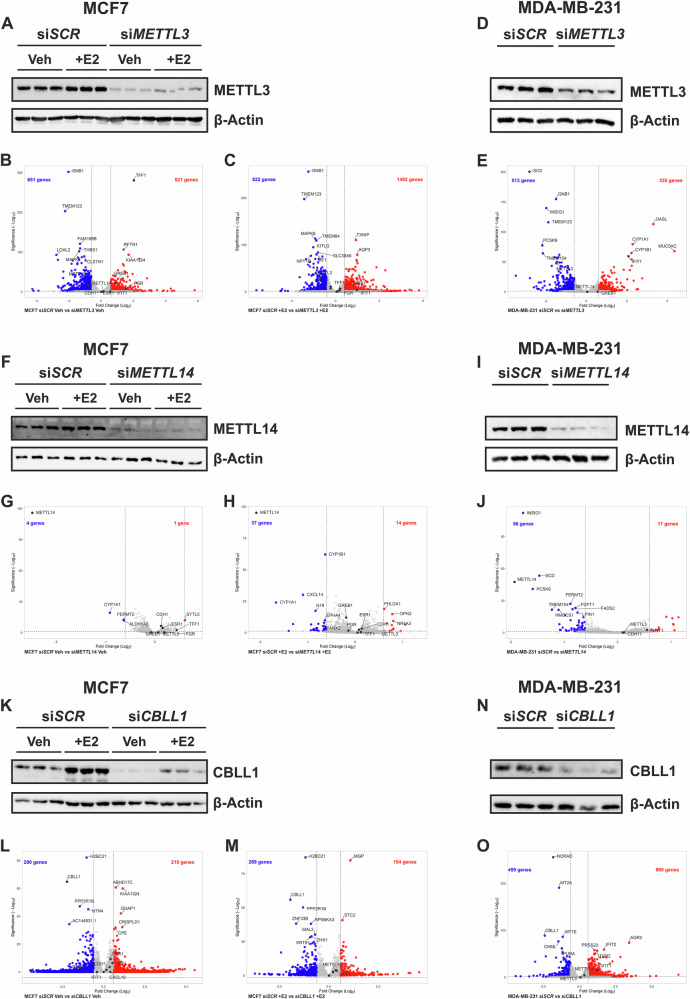
Effect of siRNA-mediated knockdown of METTL3, METTL14 and CBLL1 and E2 treatment on differential gene expression in breast cancer cell lines. siRNA-mediated knockdown of METTL3, METTL14 and CBLL1 in MCF7 and MDA-MB-231 cells (with subsequent treatment for 72 hours with vehicle or E2 in MCF7 cells) was confirmed by western blot with β-Actin loading control. Differential gene expression was analysed following RNA-seq. **A** METTL3 expression in MCF7 cells following METTL3 siRNA-mediated knockdown -/+ E2 (n = 3). **B** Differential gene expression compared between vehicle-treated siSCR control and METTL3-depleted cells (n = 3) and (**C**) between E2-treated siSCR control and METTL3-depleted cells (n = 3). **D** METTL3 expression in MDA-MB-231 cells following METTL3 siRNA-mediated knockdown (n = 3). **E** Differential gene expression compared between siSCR control and METTL3-depleted cells (n = 3). **F** METTL14 expression in MCF7 cells following METTL14 siRNA-mediated knockdown -/+ E2 (n = 3). **G** Differential gene expression compared between vehicle-treated siSCR control and METTL14-depleted cells (n = 3) and (**H**) between E2-treated siSCR control and METTL14-depleted cells (n = 3). **I** METTL14 expression in MDA-MB-231 cells following METTL14 siRNA-mediated knockdown (n = 3). **J** Differential gene expression compared between siSCR control and METTL14-depleted cells (n = 3). **K** CBLL1 expression in MCF7 cells following CBLL1 siRNA-mediated knockdown −/+ E2 (n = 3). **L** Differential gene expression compared between vehicle-treated siSCR control and CBLL1-depleted cells (n = 3) and (**M**) between E2-treated siSCR control and CBLL1-depleted cells (n = 3). **N** CBLL1 expression in MDA-MB-231 cells following CBLL1 siRNA-mediated knockdown (n = 3). **O** Differential gene expression compared between siSCR control and CBLL1-depleted cells (n = 3). Significant differential gene expression= FC ± 1.5 and adjusted p-value < 0.05.

**Fig. 4:**
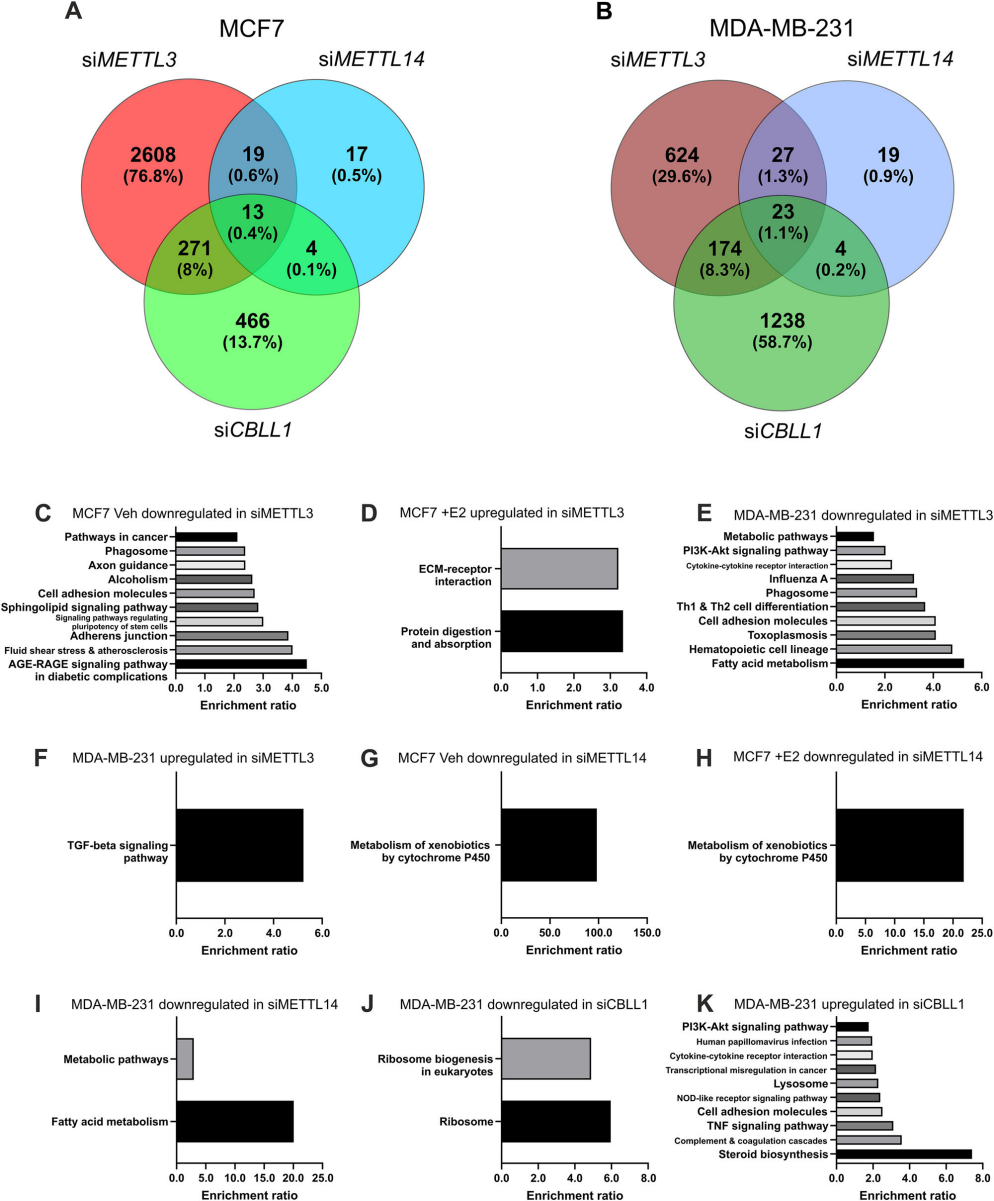
Summary of differentially expressed genes and significantly enriched KEGG pathways in breast cancer cell lines in response to siRNA-mediated METTL3, METTL14 and CBLL1 knockdown. Comparison of all DEGs in METTL3, METTL14 and CBLL1-depleted (**A**) MCF7, and (**B**) MDA-MB-231. Significantly enriched KEGG pathways associated with genes with: (**C**) downregulated expression following METTL3 depletion and vehicle treatment in MCF7; (**D**) upregulated expression following METTL3 depletion in E2 treated MCF7; (**E**) downregulated and (**F**) upregulated expression following METTL3 depletion in MDA-MB-231; (**G**) downregulated expression following METTL14 depletion and vehicle treatment in MCF7, and (**H**) downregulated expression following METTL14 depletion in E2 treated MCF7; (**I**) downregulated expression following METTL14 depletion in MDA-MB-231; (**J**) downregulated and (**K**) upregulated expression following CBLL1 depletion in MDA-MB-231. Significant gene expression= FC ± 1.5 and adjusted p-value < 0.05. All n = 3. Significant pathways FDR < 0.05.

To assess the role of METTL3, METTL14 and CBLL1 in BCa, siRNA-mediated knockdowns were performed in cell lines MCF7 and MDA-MB-231, confirmed by western blot and differential gene expression determined by RNA-seq (Fig. 3, Supplementary Figs. 3–14). It was also noted that METTL3, METTL14 and CBLL1 protein expression was upregulated by estrogen signalling in MCF7 (Fig. 3A, F and K). Comparison of differentially expressed genes revealed that METTL3, METTL14 and CBLL1 regulated distinct gene networks in MDA-MB-231 and MCF7 under estrogen depletion and with estrogen treatment (Fig. 4A–B, Supplementary Fig. 15).

METTL3 knockdown affected both the basal and estrogen-regulated transcriptome in the ERα-expressing cell line MCF7 and basal transcriptome in the TNBC cell line MDA-MB-231, including several transcription factors and genes involved in BCa carcinogenesis and progression (Fig. 3A–E). In the absence of estrogen, 1172 genes were differentially expressed in MCF7 and with E2 (10 nM) treatment 2314 genes were differentially expressed. *METTL3* regulated a large subset of E2-regulated genes. Of the genes upregulated following *METTL3* depletion in MCF7, 31% were also upregulated by E2 treatment including prototypical E2-upregulated genes such as *TFF1* (Supplementary Fig. 16). *METTL3* depletion in vehicle-treated cells resulted in ten KEGG pathways enriched in genes higher in siSCR compared with si*METTL3* including Pathways in cancer (hsa05200) and Signalling pathways regulating pluripotency of stem cells (hsa04550) (Fig. 4C). Two significantly enriched pathways were identified within the genes higher in estrogen-treated and *METTL3* depleted MCF7 compared with estrogen-treated siSCR control (Fig. 4D). These two pathways comprised Protein digestion and absorption (hsa04974) and Extracellular matrix receptor interaction (hsa04512). No pathways were enriched in the gene set upregulated by si*METTL3* in vehicle treated MCF7 or in the gene set downregulated by si*METTL3* in E2 treated MCF7. BCa and estrogen regulation associated genes were found to be altered by METTL3 knockdown, including *EGF, CDK6, PIK3R1, CDKN2B, CDKN2A, GPER1, NQO1, DNMT3B, PGR, TERT* and *SNAI1*. *METTL3* depletion in MDA-MB-231 resulted in 848 significantly differentially expressed genes (DEG), with 513 decreased and 335 increased following *METTL3* knockdown (Fig. 3E). Ten KEGG pathways were significantly enriched in the genes downregulated by *METTL3* depletion (Fig. 4E). These included Metabolic pathways (hsa01100), PI3K-Akt signalling pathway (hsa04151) and several pathways involved in innate viral response. One pathway, the TGF-beta signalling pathway (hsa04350), was significantly enriched in genes upregulated by *METTL3* depletion (FDR < 0.05; Fig. 4F).

METTL14 knockdown had modest effects on the MCF7 transcriptome. In the absence of estrogen, only five genes were significantly differentially expressed when METTL14 was depleted, whereas with estrogen 51 genes were significantly differentially expressed. KEGG pathway analysis revealed the Metabolism of xenobiotics by cytochrome P450 pathway (hsa00980) was enriched in the genes decreased when METTL14 was depleted in both vehicle and estrogen treated cells (Fig. 4G–H). A larger number of genes were significantly differentially expressed upon METTL14 depletion in MDA-MB-231 compared with MCF7 (Fig. 3F–J). Depletion of METTL14 resulted in 56 genes with significantly decreased expression and 17 genes with increased expression. In the gene set that decreased with METTL14 depletion, the significantly over-represented pathways were Metabolic pathways (hsa01100) and Fatty acid metabolism (hsa01212) (Fig. 4I). No pathways were enriched in the gene set increased upon METTL14 depletion however several of these genes were also involved in metabolism (*CYP1B1, CYP1A1, ANGPTL4, PTGES*).

Depletion of *CBLL1* in vehicle-treated MCF7 resulted in the significant differential expression of 495 genes, of which 215 were induced by CBLL1 knockdown and 280 were downregulated by *CBLL1* knockdown (including *PPL*, *TFEB* and *NTN4*) (Fig. 3K–O). In the presence of E2, depletion of CBLL1 resulted in 423 genes being differentially expressed, with 154 upregulated, and 269 genes (including *PPL*, *TFEB* and *NTN4*) downregulated (Fig. 3M). *CBLL1* depletion subtly increased the expression of *TFF1*, a prototypical estrogen-induced gene (p < 0.0001). No DEG pathways were significantly enriched following CBLL1 depletion in vehicle-treated or in E2-treated MCF7. In MDA-MB-231, 980 genes were upregulated by CBLL1 knockdown and 459 were downregulated. The pathways enriched by the genes decreased by CBLL1 depletion were Ribosome (hsa03010) and Ribosome biogenesis in eukaryotes (hsa03008) (Fig. 4J). In the genes upregulated by CBLL1 depletion the following pathways were enriched: Steroid biosynthesis (hsa00100), Complement and coagulation cascades (hsa04610), TNF signalling pathway (hsa04668), Cell adhesion molecules (CAMs) (hsa04514), NOD-like receptor signalling pathway (hsa04621), Lysosome (hsa04142), Transcriptional misregulation in cancer (hsa05202), Cytokine-cytokine receptor interaction (hsa04060), Human papillomavirus infection (hsa05165), and PI3K-Akt signalling pathway (hsa04151) (Fig. 4K).

In both MCF7 and MDA-MB-231 cell lines, METTL3 inhibition and the depletion of METTL3, METTL14 or CBLL1 dramatically altered splicing, with skipped exons being the most common differentially spliced event (Supplementary Tables 2 and 3).

### Regulation of METTL3, METTL14 and CBLL1 expression by m^6^A

**Fig. 5:**
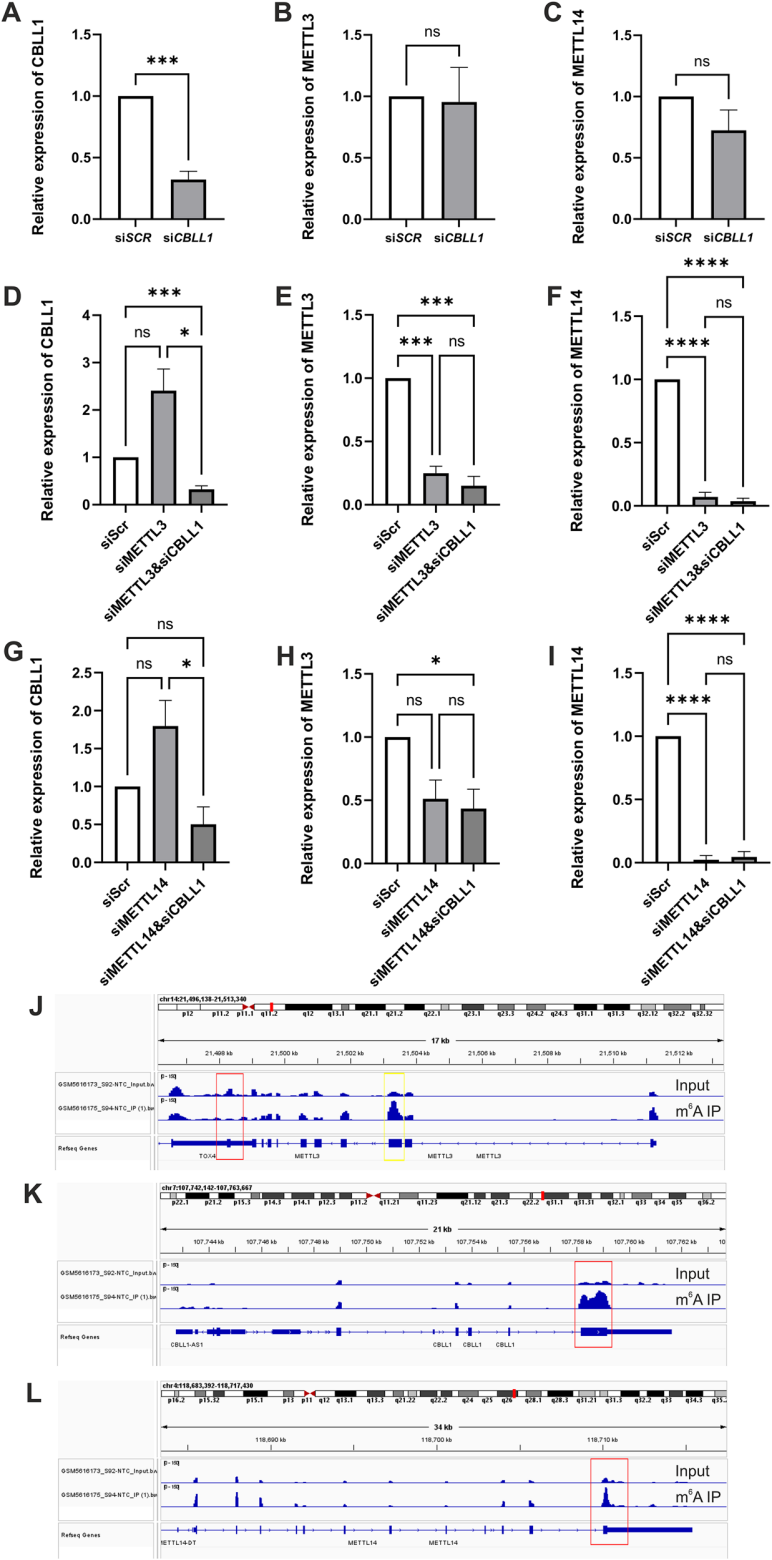
Mutual regulation of the m^6^A methylation complex components. Effect of siRNA-mediated knockdown of METTL3, METTL14 and CBLL1, both individually and in combination, on expression of METTL3, METTL14 and CBLL1 in MDA-MB-231. Quantification of expression of CBLL1, METTL3 and METTL14 determined by western blot with β-Actin loading control (n = 6) (A-I). Protein expression quantification of: (**A**) CBLL1 following siRNA-mediated knockdown of CBLL1; (**B**) METTL3 following CBLL1 siRNA-mediated knockdown; (**C**) METTL14 following CBLL1 siRNA-mediated knockdown. **D** CBLL1, (**E**) METTL3 and (**F**) METTL14 expression following siRNA-mediated knockdown of METTL3 and knockdown of METTL3 and CBLL1 in combination. **G** CBLL1, (**H**) METTL3 and (**I**) METTL14 expression following siRNA-mediated knockdown of METTL14 and knockdown of METTL14 and CBLL1 in combination. Evidence of the regulation of (**J**) METTL3, (**K**) CBLL1 and (**L**) METTL14 by m^6^A methylation in MDA-MB-231, MeRIP-seq data obtained from GEO series GSE185494. Peaks in second panel (m^6^A enriched) compared to first panel (no enrichment control) show area of m^6^A enrichment within gene. Red box highlights last exon of each gene and yellow box highlights the third exon of METTL3. * = p ≤ 0.05, ** = p ≤ 0.01, *** = p ≤ 0.001, **** = p ≤ 0.0001 by t-test or ANOVA for multiple comparisons bar charts show mean ± SEM.

METTL3 knockdown additionally reduced METTL14 protein expression, and conversely METTL14 knockdown also reduced METTL3 protein expression (Fig. 5E, F, H, I, Supplementary Figs. 10, 11 and 12), whereas mRNA expression was unchanged (Supplementary Fig. 17). This suggests auto-regulation of MTC component protein expression and stoichiometry. To determine whether the ubiquitin-ligase MTC component CBLL1 plays a role in this mutual regulation of METTL3 and METTL14 expression, the effect of combinatorial siRNA knockdown of METTL3 or METTL14 and CBLL1 on METTL14 and METTL3 expression respectively was assessed in MDA-MB-231. The addition of CBLL1 knockdown in MDA-MB-231 did not alter the effect of METTL3 or METTL14 knockdown on METTL14 and METTL3 expression (Fig. 5D-I, Supplementary Figs. 10, 11 and 12). The knockdown of CBLL1 also did not alter the expression of METTL3 or METTL14 in MDA-MB-231 or MCF7 (Fig. 5A, B and C, Supplementary Figs. 9, 13 and 14). We next assessed whether the *METTL3*, *METTL14* and *CBLL1* transcripts harboured m^6^A peaks in publicly available MeRIPseq data. Interestingly, m^6^A peaks in *METTL14* and *CBLL1* were enriched in the last exon whereas m^6^A peaks in *METTL3* were enriched in the third exon in MCF7 and MDA-MB-231 (Fig. 5J–L, Supplementary Fig. 18).

### METTL3 inhibition reduces invasion and proliferation and induces an immune gene signature in BCa cell lines

**Fig. 6:**
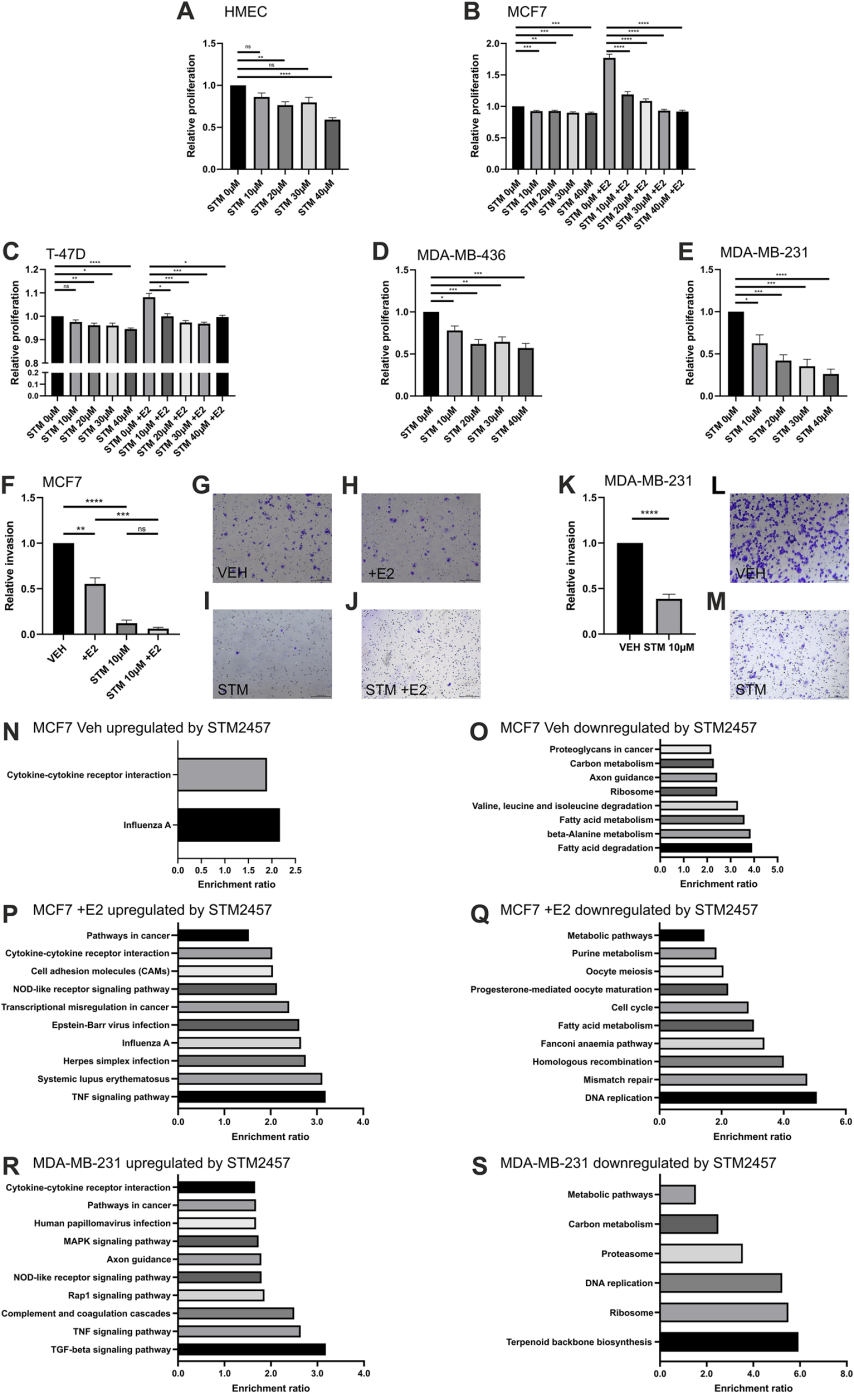
Effect of STM2457 treatment on breast cancer cell lines. Effect of increasing STM2457 treatment (STM) concentrations on proliferation in: (**A**) non-malignant HMEC (n = 6), (**B**) malignant MCF7 + /- E2 (n = 9), (**C**) malignant T-47D + /- E2 (n = 9), (**D**) malignant MDA-MB-436 (n = 9), and (**E**) malignant MDA-MB-231 (n = 9). **F** Effect of STM2457 (STM) treatment and E2 on the invasion of MCF7 (n = 8). Representative photomicrographs of MCF7 treated with (**G**) vehicle (veh), (**H**) E2, (**I**) STM2457 (STM), and (**J**) STM2457 (STM) with E2. **K** Effect of STM2457 (STM) treatment on the invasion of MDA-MB-231 (n = 8). Representative images of MDA-MB-231 treated with (**L**) vehicle (veh) and (**M**) STM2457 (STM). Significantly enriched KEGG pathways associated with genes with: (**N**) upregulated, and (**O**) downregulated expression following STM2457 treatment in MCF7; (**P**) upregulated, and (**Q**) downregulated expression following STM2457 and E2 treatment in MCF7; (**R**) upregulated, and (**S**) downregulated expression following STM2457 treatment in MDA-MB-231. * = p < 0.05, ** = p < 0.005, *** = p < 0.001, **** = p < 0.0001, ns = not significant determined by ANOVA for multiple comparisons or t-test, bar charts show mean ± SEM. Significant KEGG pathways FDR < 0.05.

The effects of pharmacological METTL3 inhibition on the phenotype, and differentially expressed and spliced transcriptome of BCa cell lines were investigated. The STM2457 small molecule METTL3 inhibitor reduced BCa cell line proliferation and invasion in both ER positive and TNBC cell lines (Fig. 6A–M). We also observed that STM2457 reduced proliferation of HMECs (Fig. 6A).

RNA-seq performed on cell line MCF7 revealed that pathways Cytokine-cytokine receptor interaction (hsa04060) and Influenza A (hsa05164) were significantly enriched in the gene set increased with STM2457 treatment (Fig. 6N). In the gene set decreased with STM2457 treatment, the pathways Axon guidance (hsa04360), Ribosome (hsa03010), Fatty acid degradation (hsa00071), Proteoglycans in cancer (hsa05205), Fatty acid metabolism (hsa01212), Valine, leucine and isoleucine degradation (hsa00280), beta-Alanine metabolism (hsa00410) and Carbon metabolism (hsa01200) were enriched (Fig. 6O).

Pathway analysis was also performed on genes significantly upregulated and downregulated by STM2457-mediated METTL3 inhibition in the presence of estrogen. Several of the pathways enriched in the gene set significantly increased with STM2457 treatment were related to cancer, viral response, and cell death (Fig. 6P). Notable pathways include TNF signalling pathway (hsa04668), Transcriptional misregulation in cancer (hsa05202), Pathways in cancer (hsa05200) and Cytokine-cytokine receptor interaction (hsa04060). Several immune related genes were common between MCF7 vehicle and E2 treated, including genes *CXCL10*, *IFIT1* and *DDX58* (*RIG1*). In the gene set decreased with STM2457 treatment, pathways related to cell division and metabolism were significantly enriched (Fig. 6Q) and included Metabolic pathways (hsa01100) and Cell cycle (hsa04110). Many of these metabolic pathways were also significantly enriched with METTL3 inhibition without estrogen treatment (Fig. 6O).

Fewer genes were significantly differentially expressed by STM2457 treatment of MDA-MB-231 compared to MCF7 (Supplementary Fig. 19). Several cancer-associated pathways were significantly enriched in the gene set induced by STM2457, including MAPK signalling pathway (hsa04010), TGF-beta signalling pathway (hsa04350) and Pathways in cancer (hsa05200) (Fig. 6R). There were also several pathways associated with immune and stress response: NOD-like receptor signalling pathway (hsa04621), TNF signalling pathway (hsa04668), Human papillomavirus infection (hsa05165), Complement and coagulation cascades (hsa04610) and Cytokine-cytokine receptor interaction (hsa04060). In the genes decreased by STM2457 in MDA-MB-231, over-represented genes were associated with the following KEGG pathways: Ribosome (hsa03010), Metabolic pathways (hsa01100), DNA replication (hsa03030), Terpenoid backbone biosynthesis (hsa00900), Carbon metabolism (hsa01200) and Proteasome (hsa03050) were enriched (Fig. 6S). Within the DNA replication pathway is the gene Proliferating Cell Nuclear Antigen (PCNA) a well-defined marker of cell division^35^. In the ribosome pathway, 58 of the 153 ribosome-associated genes decreased with STM2457 treatment.

To give further insight into the expression of *METTL3* in BCa tissue samples, the TCGA RNA-seq data was utilised (n = 1078). The IL-17 signalling pathway (hsa04657) was significantly enriched in the gene set that was increased when *METTL3* expression was low (Supplementary Fig. 20).

## Discussion

In this large BCa patient cohort study, we found that higher nuclear CBLL1 and METTL14 expression was significantly associated with several favourable prognostic factors, most notably lower grade, better NPI prognostic group, ER and PR positivity, and non-TNBC. Kaplan-Meier survival analysis revealed that higher nuclear METTL14 and CBLL1 expression were both significantly associated with longer BCSS, TTDM and DFS. However, only CBLL1 was also found to be an independent prognostic indicator of BCSS, TTDM and DFS when considering the potential confounding variables of nodal stage, tumour size, patient age, ER status and LVI.

These findings were consistent with previous studies that reported a tumour suppressor role for CBLL1 in BCa, including association between higher CBLL1 expression and reduced proliferation and migration in BCa cells^36^, and better relapse-free survival and OS in BCa patients (n = 134)^28^. In agreement with our findings that increased METTL14 expression correlated with more favourable clinical prognostic factors and outcomes, TCGA GDC transcriptomic data showed decreased *METTL14* expression in tumour tissue compared with normal breast tissue. Additionally, two studies have reported reduced METTL14 protein expression in BCa compared to normal breast tissue^23,37^.

Further supporting the role of m^6^A in BCa, we have previously shown that low mRNA and protein expression of m^6^A demethylase ALKBH5 was associated with poorer clinical outcomes and prognosis^38^. Additionally, the other known components of the m^6^A MTC have also been implicated in BCa. Downregulated ZC3H13 and upregulated VIRMA have been shown to correlate with poor prognosis in BCa^37,39^. RBM15 has been found to be elevated in basal-like BCa and TNBC and associated with poorer outcomes, likely through regulation of serine and glycine metabolism^40^. WTAP has been shown to be predominantly associated with a cancer-promoting role in BCa^41,42^.

Despite higher METTL3 expression being significantly associated with lower tumour grade, ER and PR positivity, and non-TNBC status, METTL3 expression did not correlate with survival outcomes in our patient cohort, when considering the cohort as a whole. *METTL3* expression also did not correlate with survival in TCGA data. However, higher nuclear METTL3 expression was significantly associated with decreased BCSS, TTDM and DFS in TNBC patients. High nuclear METTL3 expression was also an independent prognostic indicator of poorer BCSS, TTDM and DFS independent of patient age, ER status, LVI, nodal stage, tumour grade and size in both the whole cohort and the TNBC sub-cohort. Therefore, the associations between increased METTL3 expression with favourable clinicopathological variables are likely due to the association of METTL3 expression with estrogen signalling. Similarly, METTL3 expression, together with METTL14 and CBLL1, was found to be positively correlated with the expression of proteins associated with better BCa prognosis such as BRCA1, ERα and FOXA1^43–45^.

The individual siRNA depletion of *METTL3* and *METTL14* also reduced expression of METTL14 and METTL3 protein, respectively, without altering mRNA expression. Similarly, Chelmicki and colleagues reported that *METTL3* knockout resulted in loss of METTL14 protein, and *METTL14* knockout reduced METTL3 protein expression in mouse embryonic stem cells^46^. Similar results were also obtained in *Arabidopsis*, demonstrating that MTA and MTB (METTL3 and METTL14 homologues, respectively) have interdependent effects on protein accumulation and localisation^47^. The exact mechanism of this evolutionarily conserved reciprocal regulation of MTC components remained unknown. For this reason, we assessed whether the CBLL1 ubiquitin-ligase component of the MTC may play a role in sensing and regulating the relative stoichiometry of the METTL3 and METTL14 MTC components. To do this we conducted individual and combinatorial knockdown of METTL3 or METTL14 with CBLL1. Knockdown of CBLL1 did not affect the reciprocal mutual regulation of METTL3 and METTL14, suggesting an alternative mechanism, potentially involving m^6^A regulation of METTL3 and METTL14 expression. To address this we next assessed publicly available MeRIPseq data from BCa cell lines to evaluate whether the *METTL3*, *METTL14* and *CBLL1* transcripts harboured m^6^A. *METTL3*, *METTL14* and *CBLL1* transcripts were found to have m^6^A peaks. Thus, knockdown of either METTL3 or METTL14 would impact MTC function, reducing global m^6^A, including of *METTL3* and *METTL14* transcripts, thereby affecting their protein expression. It is also possible that when one component of the catalytic METTL3:METTL14 heterodimer was depleted, then the heterodimer was unable to form, and the other component became more susceptible to protein degradation. Further studies are warranted to determine the exact mechanism of MTC regulation and the functional effect of the m^6^A peak within the third exon of *METTL3* transcripts.

RNA-seq analyses following the knockdown of METTL3, METTL14 and CBLL1 revealed that each regulates distinct gene networks and pathways in the BCa cell lines examined here. Several KEGG pathways including Pathways in cancer (hsa05200) were enriched in genes higher in siSCR Veh compared to si*METTL3* Veh. METTL3 depletion had distinct functions and more pathway-targeted effects in the absence of E2 signalling in MCF7. Multiple KEGG pathways were significantly enriched in the genes downregulated by METTL3 depletion in MDA-MB-231 suggesting selective transcriptional effects, not transcriptome-wide, non-specific effects. These included Metabolic pathways (hsa01100), PI3K-Akt signalling (hsa04151) and several pathways involved in viral response. This is consistent with MeRIP-seq analysis reported by Li and colleagues that showed m^6^A modification peaks were enriched in the Metabolic, TNF signalling, and PI3K-Akt–related transcripts in glioma stem cells^48^. The reduction in the pro-survival and pro-proliferative PI3K-Akt signalling pathway under METTL3 depletion may underlie the poor prognosis associated with high METTL3 expression in the TNBC patient cohort in this study. Indeed, the PI3K-Akt signalling pathway is often activated in BCa through PIK3CA, AKT1 or MTOR activation and/or loss of PTEN and INPP4B, and therapeutics targeting this pathway are being developed^49,50^. Interestingly, several immune pathways were enriched in the gene set downregulated by *METTL3* depletion in contrast to the inhibition of METTL3 by STM2457 in which immune pathways were increased. Despite the immune-related pathways being enriched, viral RNA response genes such as *OASL*, *IFIT1*, *IFIT2* and *IFIT3* were upregulated following METTL3 depletion in MDA-MB-231. Inconsistencies between METTL3 inhibition and depletion may be the result of METTL3 depletion resulting in a residual level of functional METTL3, disruption of the MTC formation and function or due to non-m6A methyltransferase roles of METTL3^51,52^.

Of the 51 genes commonly significantly downregulated by METTL3 depletion in both MCF7 and MDA-MB-231, several have been implicated in BCa including *ZNF652, GPER1*, and *STC1*. *ZNF652* encodes a transcription factor reported to promote expression of genes associated with cancer development and progression^53^. GPER1 (G protein-coupled estrogen receptor) binds E2 and rapidly induces non-genomic E2 effects and its expression has recently been found to be associated with poor outcomes in TNBC^54,55^. GPER1 has been reported to interact with and possibly induce ERα36, a truncated and transcriptionally inactive isoform of ERα^56,57^. *STC1* transcription has already been reported to be upregulated indirectly by m^6^A^21^. *STC1* is reported to promote BCa growth and metastasis and may also promote homologous recombination-mediated DNA damage repair by recruiting BRCA1^58,59^.

In contrast to METTL3 and CBLL1 depletion, METTL14 depletion affected the expression of few genes reinforcing the importance of METTL3 as the catalytic component of the MTC. Indeed, the reduction of METTL3 observed following METTL14 depletion did not induce the transcriptional changes observed following direct METTL3 depletion. One explanation is that the downregulation of METTL3 following METTL14 depletion may have been insufficient to induce the transcriptional changes or may have resulted in the reduction of METTL3 protein that, due to sub-cellular distribution and function, did not function to influence the transcriptome. METTL14-regulated DEGs included both oncogenic and tumour suppressor genes and demonstrate a role of METTL14 in regulating metabolism in breast cancer. These transcriptomic effects may be due to both m^6^A changes and the role of METTL14 in chromatin regulation^60^.

RNA-seq analysis of CBLL1 depleted MCF7 cells revealed a number of DEGs associated with BCa, including *PPL, TFEB* and *NTN4*, that offer a mechanistic basis for the association of CBLL1 with better outcomes. *PPL*, *TFEB* and *NTN4* expression levels were reduced by CBLL1 depletion. Downregulated *PPL* (periplakin) expression has been associated with cancer progression in a number of cancer types^61–63^, and has been implicated in brain metastasis in TNBC^64^. The *TFEB* gene encodes the transcription factor EB, a master regulator of several essential cellular processes, including cellular differentiation, autophagy, cellular energy metabolism, lysosome regulation, and immune response^65–69^. In early stage BCa, high TFEB expression has been associated with a poorer prognosis in patients^70^, although another study found that increased TFEB expression reduced breast tumour development through modulation of tumour-associated macrophages, and concluded that TFEB activation represented a promising therapeutic approach^71^. *NTN4* was found to be downregulated following the siRNA-mediated knockdown of CBLL1, consistent with our findings that low CBLL1 correlates with poorer outcomes. It has been shown previously that low NTN4 expression was associated with poorer survival in BCa and associated with the infiltration of numerous immune cell types^72^.

In MDA-MB-231, CBLL1 knockdown resulted in enriched pathways consistent with CBLL1 being a positive prognostic indicator. In the genes upregulated by CBLL1 depletion, the Steroid biosynthesis, Complement and coagulation cascades, TNF signalling pathway, Cell adhesion molecules (CAMs), NOD-like receptor signalling pathway, Lysosome, Transcriptional misregulation in cancer, Cytokine-cytokine receptor interaction, Human papillomavirus infection and PI3K-Akt signalling pathways were enriched. The Ribosome and Ribosome biogenesis in eukaryotes pathways were enriched by genes downregulated following CBLL1 depletion, indicating a potential reduction in protein translation. As with METTL3 inhibition by STM2457, we observed an increase in immune signalling following CBLL1 depletion, further suggesting m^6^A mediation.

Our data suggests a role for these m^6^A regulators in modulating E2-regulated gene expression. METTL3, METTL14 and CBLL1 protein expression was upregulated by estrogen in MCF7. Both siRNA-depletion and pharmacological inhibition of METTL3 also resulted in the upregulation of prototypical estrogen-regulated genes, such as *TFF1*. This finding is supported by a recent study by Wan and colleagues that determined a role for METTL3-dependent m^6^A methylation in balancing estrogen and progesterone signalling^73^. They determined that conditional knockout of METTL3 causes hyperactivation of the estrogen response by stabilising mRNAs in murine uterine tissue^73^. This suggests that METTL3 functions as a transcriptional “dimmer switch” of basal expression of estrogen-regulated genes. Thus, high METTL3 expression could downregulate E2-driven proliferation and may explain why increased METTL3 expression is not significantly associated with a poorer prognosis in hormone receptor positive BCa. Similarly, CBLL1 depletion subtly increased the expression of *TFF1*, indicating this may be an m^6^A-mediated mechanism. However, another study found that through directly binding ERα and competing with coactivators, CBLL1 acts as a co-repressor of ERα^36^.

METTL3, METTL14 and CBLL1 depletion had an extensive effect on splicing in both MCF7 and MDA-MB-231 including many genes implicated in BCa. This result is consistent with another study that found METTL3 promoted alternative splicing events associated with the malignant phenotype of BCa, both directly through m^6^A and by the regulation of transcription factors such as MYC which led to decreased SRSF11 splicing factor protein levels^74^. We observed no significant change to the *SRSF11* transcript level following METTL3 depletion. METTL3 inhibition by STM2457 has also been found to alter splicing in prostate cancer^32^, and may have a role in supressing tumorigenesis.

Treatment with the STM2457 small molecule inhibitor reduced cellular proliferation and invasion in both ER positive and TNBC cell lines, but also reduced proliferation of primary mammary epithelial cells (HMECs). This finding is in line with other studies that reported reduced proliferation and invasion of BCa cell lines with the knockdown of METTL3 and increased invasion and migration upon METTL14 overexpression^19,22,74,75^. Another study supporting our findings demonstrated that STM2457 treatment reduced growth and had a synergistic effect with carboplatin and Olaparib treatment in both MDA-MB-231 and a TNBC patient-derived organoid^76^. STM2457 treatment significantly altered the transcriptome and mRNA splicing, and several mechanisms that could reduce cancer cell growth were identified. Another potential mechanism is that the reduction or absence of m^6^A is hindering effective processing of RNA and protein translation, as demonstrated by the dramatic changes in transcripts and splicing alterations, which is impeding cellular processes and preventing proliferation and migration. We also demonstrated that STM2457 induces immune and inflammatory pathways, particularly those in response to viral infection. Although immune and inflammatory signalling activation is often associated with BCa progression and poor patient outcomes, the decrease in proliferation and invasion observed in phenotypic studies suggests that these pathways are not acting in a pro-tumourigenic manner, although the tumour microenvironment is critical for immune-related features.

The role of m^6^A in the innate immune system has been previously explored in several studies^77–81^. It was shown in foetal murine hematopoietic stem cells that loss of METTL3 and m^6^A resulted in the formation of endogenous double-stranded RNAs (dsRNAs) which activated an aberrant innate immune response^82^. It is therefore likely that m^6^A depletion is resulting in the activation of the antiviral response in BCa. Indeed, recently, inhibition of METTL3 with STM4257 and another METTL3 inhibitor (STM3006) was shown to stimulate an interferon response, augment antigen-dependent killing of cancer cells by T-cells in vitro and exhibit far greater in vivo pre-clinical activity when in combination with anti-PD1 therapy^34^. The analysis of TCGA RNA-seq data revealed evidence of an association between METTL3 and immune response in BCa tumour specimens, as low METTL3 expression correlated with an increased expression of genes involved in immune-related pathways. Further studies into the potential functional interactions between m^6^A and immune-targeted therapies in BCa are therefore warranted.

Further work is required to understand the distinct cell line and context specific transcriptional networks regulated by these MTC components. The established E3 ubiquitin ligase function of CBLL1 and the emerging m^6^A-independent functions of METTL3 and METTL14 provide potential explanations for the distinct differential gene expression observed between the different siRNA knockdowns^25,51,52,60^. However, the distinct gene expression profiles observed between MDA-MB-231 and MCF7 in estrogen depleted and estrogen treated conditions, indicate BCa subtype and endocrine conditions have a dramatic influence on the molecular consequences of METTL3, METTL14 and CBLL1 activity. Critically, the examination of transcript m^6^A levels by methods such as MeRIPseq or Nanopore Direct RNA sequencing (DRS), would enable the effects observed here due to knockdown and METTL3 inhibition, such as the modulation of E2 signalling, to be attributed to either the absence of m^6^A on specific transcripts, indirect m^6^A effects or to the other non-m^6^A roles of these proteins. Further work is now required to determine the effects of METTL3 inhibition in vivo. STC-15, the METTL3 inhibitor currently undergoing clinical trial, like STM2457, also binds the SAM pocket of METTL3^34^. However, STC-15 has improved potency, oral bioavailability and metabolic stability and future in vivo studies may benefit from utilizing STC-15 over STM2457.

In conclusion, our findings suggest important clinical and mechanistic roles for the m^6^A methyltransferase complex proteins, METTL3, METTL14 and CBLL1, in the regulation of gene expression and alternative splicing in BCa and estrogen signalling. IHC analysis of ~2500 BCa tumour specimens revealed an association of increased METTL14 and CBLL1 with favourable clinical outcomes in BCa and increased METTL3 expression with poor clinical outcomes in hormone receptor negative BCa. Transcriptomic analysis of METTL3, METTL14 and CBLL1 depletion in BCa cell lines revealed the regulation of distinct transcriptional networks and demonstrated that METTL3 modulates estrogen signalling and has BCa-subtype specific roles. The inhibition of the methyltransferase activity of METTL3 reduced the invasive and proliferative phenotype of BCa cell lines and increased the expression of immune-related pathways. These results highlight the promising potential of METTL3 inhibitors as an effective therapeutic option in BCa, particularly TNBC. Crucially, the safety profile of STC-15, a derivative of STM2457, from the ongoing phase 1 trial will determine the potential of therapeutically targeting m^6^A to benefit patients with BCa.

## Methods

### Bioinformatic analysis of *METTL3*, *METTL14* and *CBLL1* in clinical specimens

*METTL3*, *METTL14* and *CBLL1* expression and gene alterations were analysed in The Cancer Genome Atlas (TCGA) breast invasive carcinoma cohort (PanCancer Atlas, n = 996)^83^ using the cBioPortal for Cancer Genomics (retrieved January 2024)^84,85^. Analysis of *METTL3*, *METTL14* and *CBLL1* expression in the GDC TCGA Breast Cancer patient cohort^83^ was carried out using the UCSC Xena Functional Genomics Browser (n = 1284)^86^. The Kaplan-Meier Plotter online tool^87^ was used to investigate the association of *METTL3*, *METTL14* and *CBLL1* expression with patient survival. RNA-seq data utilising best performing cut-off for dichotomisation for OS was used (n = 2,976). TCGA RNA-seq data was utilised to perform differential gene expression analysis based on METTL3 expression in primary female BCa samples (n = 1078). Count data, generated by HTSeq, was obtained from the Genomics Data Commons and was normalised within the Deseq2 package and quartiles assigned for METTL3 expression. DESeq2 was utilized to determine the differentially expressed genes between Quartile 1 (breast cancer with low *METTL3* expression) and Quartile 4 (breast cancer with high *METTL3* expression)^88^. Methylated RNA Immunoprecipitation Sequencing (MeRIP) data was obtained from Gene Expression Omnibus (GEO) for MCF7 (ER positive BCa cell line) Series GSE143441^89^ and MDA-MB-231 (TNBC cell line) Series GSE185494^90^ and viewed using Integrative Genomics Viewer (IGV)^91^.

### Immunohistochemistry of BCa tissue specimens

A breast tumour tissue microarray (TMA) was constructed from a well-characterised Nottingham University Hospitals (NUH) NHS Trust patient cohort. The cohort comprises surgically removed tumour tissue from over 2800 BCa patients diagnosed between 1990 and 2006. All patients were neoadjuvant treatment-naïve at the time of surgery and all patient clinical data were maintained prospectively.

IHC was carried out using the Novolink Max Polymer Detection System (Leica Biosystems, UK) as previously described^92^. Sections (4 µm) were cut from the TMA blocks and subsequently stained with primary antibodies: anti-METTL3 (ab195352 PUR; Abcam, UK, 1:500), anti-METTL14 (ab220030; Abcam, UK, 1:3000), anti-CBLL1 (NBP1-83589; Novus Biologicals, USA, 1:100). All primary antibodies were incubated at room temperature for one hour. Following staining, all slides were scanned at high resolution with the Nanozoomer (Hamamatsu Photonics, UK). The siRNA-mediated knockdowns performed in this study support the specificity of these antibodies.

Nuclear staining was assessed using the semi-quantitative histo-score (H-score) system^93^. Staining was performed on intact cores with tumour coverage of >10% of the core. A second scorer independently assessed a minimum of 10% of all specimens in order to validate all H-scores, confirmed with concordance of >0.8 via intraclass correlation.

Protein expression scores were dichotomised into high and low expression groups by median H-score and correlated with clinicopathological parameters and patient outcomes. METTL3 staining was divided into H-score groups of low ≤105 and high ≥106, METTL14 into H-score groups of low ≤120 and high ≥121, and CBLL1 into H-score groups of low ≤70 and high ≥71. Protein expression was also correlated with markers previously studied^94–97^.

Ethics approval for the construction of the TMA for samples collected from 1990-1998 was granted by the Nottingham Research Ethics Committee 2 (Reference title: Development of a molecular genetic classification of breast cancer), with ethics approval for samples collected from 1998-2006 granted by the North West Greater Manchester Central Research Ethics Committee (Reference title: Nottingham Health Science Biobank; reference number: 15/NW/0685). The Human Tissue Act and Helsinki Declaration of Human Rights were strictly observed, and the General Data Protection Regulation (GDPR) was applied.

### Cell lines and culture conditions

Human Mammary Epithelial Cells (HMECs), ER-expressing malignant cell lines MCF7 and T-47D, and ER-negative malignant cell lines MDA-MB-231 and MDA-MB-436 were utilised in this study. Cell line identity was confirmed by genotyping or STR profiling. All cells were cultured at 37 °C and 5% CO_2_.

MCF7, T-47D and MDA-MB-231 were cultured in RPMI-1640 medium with L-glutamine supplemented with 100 µg/mL penicillin, 100 µg/mL streptomycin, 1 mM sodium pyruvate (all from Gibco® by Life Technologies, USA), and 10% heat-inactivated foetal bovine serum (FBS) (Sigma-Aldrich, USA). For estrogen treatment experiments, cells were grown in phenol-red free RPMI-1640 media supplemented with 100 µg/mL penicillin, 100 µg/mL streptomycin, 1 mM sodium pyruvate, 2 mM L-glutamine (all from Gibco by Life Technologies, USA), and 10% charcoal-stripped FBS with β-estradiol (E2) (10 nM) (Tocris Biosciences, UK) or DMSO as a vehicle control. HMEC cells were cultured in HuMEC Basal Serum-Free Medium with addition of the HuMEC Supplement Kit (Gibco by Life Technologies, USA). MDA-MB-436 cells were cultured in DMEM F12 media supplemented with HEPES, 100 µg/mL penicillin, 100 µg/mL streptomycin and 1 mM sodium pyruvate (Gibco by Life Technologies, USA).

### siRNA-mediated functional depletion of *METTL3*, *METTL14* and *CBLL1* and pharmaco-inhibition of METTL3

siRNA-mediated depletion of *METTL3*, *METTL14* and *CBLL1* was performed using DharmaFECT 1 Transfection Reagent (GE Dharmacon, USA) and siRNA targeting *METTL3* (SMARTpool: ON-TARGETplus *METTL3* siRNA L-005170-02-0010; GE Dharmacon, USA), *METTL14* (SMARTpool: ON-TARGETplus *METTL14* siRNA L-014169-02-0010; GE Dharmacon, USA) and *CBLL1* (SMARTpool: ON-TARGETplus *CBLL1* siRNA L-007069-00-0005; GE Dharmacon, USA), respectively, following the manufacturer’s instructions at a final concentration of 20 nM siRNA. The ON-TARGETplus non-targeting control siRNA (D-001810-10-20, GE Dharmacon, USA) was employed as a negative control (scrambled control, siSCR). Following the first transfection, the media was replaced and a second transfection performed 48 hours later. Cellular RNA or protein was harvested after a further 72 hours. MCF7 was additionally treated with E2 (10 nM) (Tocris Biosciences, UK) or DMSO as a vehicle control at the second transfection.

Functional inhibition of METTL3 was conducted in the MCF7 and MDA-MB-231 cell lines using the METTL3 inhibitor STM2457 (MedChemExpress, USA). The cells were treated with either STM2457 (10 μM) or DMSO (vehicle) for 6 days. MCF7 was additionally treated with E2 (10 nM) (Tocris Biosciences, UK) or DMSO as a vehicle control.

### Gene expression analysis and western blotting

For mRNA and protein expression analysis, BCa cells were treated as described above. RNA isolation was carried out as per the manufacturer’s protocol using the GenElute™ Mammalian Total RNA Miniprep Kit with on-column DNase treatment (Sigma-Aldrich, USA). cDNA was synthesised using the qScript™ cDNA Synthesis Kit (Quanta Biosciences, USA). mRNA expression analysis was performed using real-time quantitative polymerase chain reaction (RT-qPCR) with TaqMan™ Fast Advanced Master Mix and Taqman™ Real-Time PCR probes (Thermo Fisher Scientific™, USA) and carried out in a CFX Connect Real-Time PCR Detection System (Bio-Rad Laboratories, USA). The Taqman™ probes used in this study were *β-Actin* (Hs01060665_g1), *METTL3* (Hs00219820_m1) and *METTL14* (Hs00383340_m1) from Thermo Fisher Scientific (Waltham, USA). Relative mRNA expression was calculated using the Pfaffl method^98^ and analysed relative to *β-Actin*.

Total protein analysis by western blotting was carried out as previously described^32,99^. The following antibodies were used in this study: anti-METTL3 (ab195352 PUR; Abcam, UK; 1:10,000), anti-METTL14 (ab220030; Abcam, UK; 1:1,000), anti-CBLL1 (NBP1-83589; Novus Biologicals, USA; 1:1,000), and anti-β-Actin (sc-130657; Santa Cruz, USA, 1:50,000 or MA5-15739; Invitrogen, USA, 1:10,000). Goat anti-mouse (ab97023; Abcam, UK, 1:10,000–50,000 or Sc-2005; Santa Cruz, USA, 1:10,000–50,000) and goat anti-rabbit (ab6721; Abcam, UK, 1:10,000-50,000 or Sc-2004; Santa Cruz, USA, 1:10,000-50,000) secondary antibodies were used. Western blots from all experimental repeats and full, uncropped and annotated western blot images are available in Supplementary Figs. 3–14.

### RNA-seq and splicing analysis of siRNA depletion or pharmaco-inhibition of METTL3

RNA-seq analysis (Novogene, UK) was performed on MCF7 and MDA-MB-231 BCa cells transfected with siSCR control, si*METTL3*, si*METTL14* or si*CBLL1*. Additionally, RNA-seq was performed on MCF7 and MDA-MB-231 cells treated either with vehicle (DMSO) or 10 μM STM2457. MCF7 experiments were also performed in vehicle (DMSO) or estrogen (10 nM E2) conditions. Analysis of the obtained Fastq files was carried out as previously described^99^. Genes were determined significant by fold change (FC) ± 1.5 and false discovery rate (FDR) < 0.05 and are listed in Supplementary File 1. Differential alternative splicing was also assessed using replicate multivariate analysis of transcript splicing (rMATS, version 3.2.5)^100^. Biologically and statistically significant differential alternative splicing was determined by difference in percentage spliced in (dPSI) ≥ 5% and false discovery rate (FDR) < 0.05. The Web-Based Gene Set AnaLysis Toolkit (WebGestalt) was utilised to identify significantly enriched Kyoto Encyclopaedia of Genes and Genomes (KEGG) pathways and weighted set cover filter applied^101^. Venny 2.1 (https://bioinfogp.cnb.csic.es/tools/venny/) was utilised to produce Venn diagrams to compare gene sets^102^ and the VolcaNoseR ShinyApp (https://huygens.science.uva.nl/VolcaNoseR/) was used to prepare volcano plots of significantly differentially expressed genes. Significantly differentially expressed gene sets were compared using Venny 2.1.0 (csic.es). All RNA-sequencing data reported here is available from NCBI-GEO (www.ncbi.nlm.nih.gov/geo) under the following accession numbers: GSE195532 (si*METTL3* in MCF7), GSE195487 (si*METTL14* in MCF7), GSE234571 (si*METTL3* and si*METTL14* in MDA-MB-231), GSE271326 (si*CBLL1* in MCF7), GSE271330 (si*CBLL1* in MDA-MB-231) and GSE234322 (STM2457 treatment of MCF7 and MDA-MB-231).

### Phenotypic analysis

The effect of METTL3 inhibition on the proliferation of HMEC, MCF7, T-47D, MDA-MB-436 and MDA-MB-231 over six days was determined. Following plating, cells were treated 24 hours later with varying concentrations of STM2457 (0 µM, 10 µM, 20 µM, 30 µM and 40 µM) in the cell-appropriate medium, with media and treatment replaced after 72 hours. E2 (10 nM) or DMSO vehicle control conditions were also performed for MCF7 and T-47D. After a further 72 hours, cellular proliferation was measured using the CyQUANT™ Direct Cell Proliferation Assay (Invitrogen, USA) as per the manufacturer’s instructions, and the relative DNA content calculated.

Changes in cellular invasion following STM2457 treatment were assessed in cell lines MCF7 and MDA-MB-231. Cells were pre-treated for five days prior to the assay with either STM2457 (10 µM) or DMSO vehicle control. In MCF7, +/- E2 (10 nM) conditions were also applied. Changes in cellular invasion following STM2457 treatment were assessed using a Matrigel (Corning, USA) invasion assay as previously described^99^. 5 ×10^5^ MCF7 cells and 2.5 ×10^5^ MDA-MB-231 cells were plated prior to fixing at 24 hours. A total of 12 fields of view were systematically imaged per well using an inverted microscope (Leica, Germany), with the resultant images subsequently manually assessed to quantify the total number of cells that had invaded, with relative invasion calculated compared to vehicle control.

### Statistical analysis

Statistical analysis was carried out using GraphPad Prism (GraphPad Software Inc., USA) and SPSS v26.0 (IBM, USA) statistical software. T-tests were carried out for the comparison of two means. For the comparison of multiple means a one-way analysis of variance (ANOVA) was performed. The median H-score was utilised to dichotomise protein expression into low and high expression groups, with 15-year breast cancer-specific survival (BCSS), disease-free survival (DFS) or time to distant metastasis (TTDM) used as an endpoint. Correlations with clinicopathological parameters were analysed by χ² test (asymptomatic significance, 2-sided) and the statistical significance of Kaplan Meier estimates calculated using the log-rank (Mantel-Cox) test. Combined multivariate Cox regression analysis (or proportional hazards model) was utilised to assess the prognostic independence of a marker from established prognostic indicators. Correlation between METTL3, METTL14 and CBLL1 with other previously assessed markers was determined by Spearman’s rank correlation coefficient. Graphs were generated with GraphPad Prism 9 (GraphPad Software, USA) and data were expressed as mean ± standard error of the mean (SEM). A p value of <0.05 was considered statistically significant.

#### Ethics approval and consent to participate

This study was reviewed and approved by the Nottingham Research Ethics Committee, (approval # REC202313), and the research ethics committee of the University of Nottingham School of Veterinary Medicine and Science (approval #3483 211102). The General Data Protection Regulation (GDPR) was applied, and informed consent obtained. The Helsinki Declaration of Human Rights was strictly observed.

## Supplementary information

Supplementary figures

Supp data 1

## Data Availability

Data are available upon reasonable request. All RNA-sequencing data reported here is available from NCBI-GEO (www.ncbi.nlm.nih.gov/geo) under the following accession numbers: GSE195532 (siMETTL3 in MCF7), GSE195487 (siMETTL14 in MCF7), GSE234571 (siMETTL3 and siMETTL14 in MDA-MB-231), GSE271326 (siCBLL1 in MCF7), GSE271330 (siCBLL1 in MDA-MB-231) and GSE234322 (STM2457 treatment of MCF7 and MDA-MB-231).

## Code availability

Example code used to analyse the data (which is available in GEO accession numbers: GSE195532 (siMETTL3 in MCF7), GSE195487 (siMETTL14 in MCF7), GSE234571 (siMETTL3 and siMETTL14 in MDA-MB-231), GSE271326 (siCBLL1 in MCF7), GSE271330 (siCBLL1 in MDA-MB-231) and GSE234322 (STM2457 treatment of MCF7 and MDA-MB-231). Trimgalore: trim_galore -q 30 --fastqc --paired sample.R1.fastq.gz sample.R2.fastq.gz. STAR alignment: STAR --genomeDir /starindexGrch38.83_star273a/ --sjdbGTFfile /starindexGrch38.83_star273a/Homo_sapiens.GRCh38.83.gtf --runThreadN 6 --readFilesIn sample_1_val_1.fq.gz sample_2_val_2.fq.gz --readFilesCommand zcat --outSAMtype BAM SortedByCoordinate --chimSegmentMin 20 --chimOutType WithinBAM --outFileNamePrefix sample_star_output; To trim reads for rMATs. python trimFastq.py sample_1_val_1.fq.gz 145_sample_1_val_1.fq.gz 145. rMATs: python2.7 /home/MATS.3.2.5/RNASeq-MATS.py -s1 -s2 -gtf Homo_sapiens.GRCh38.83.gtf -bi /home/star_v271a/ -novelSS 1 -len 145 -t paired -o sample_output.
