# Supplementary material for: Functional and clinical significance of the RNA m6A methyltransferase complex in breast cancer
Source: NPJ Breast Cancer. 2025 Nov 27;11:147. doi: 10.1038/s41523-025-00861-5 (PMC12722736; doi:10.1038/s41523-025-00861-5)
Supplement: Supplementary file 1 — Supplementary figures [file 41523_2025_861_MOESM1_ESM.pdf]

A

	Amplification	Deep Deletion	Missense mutation	Truncating mutation	No genetic alteration
METTL3	7 (0.7%)	2 (0.2%)	2 (0.2%)	1 (0.1%)	984 (98.8%)
METTL14	5 (0.5%)	0 (0%)	3 (0.3%)	1 (0.1%)	987 (99.1%)
CBLL1	6 (0.6%)	1 (0.1%)	1 (0.1%)	0 (0%)	988 (99.2%)

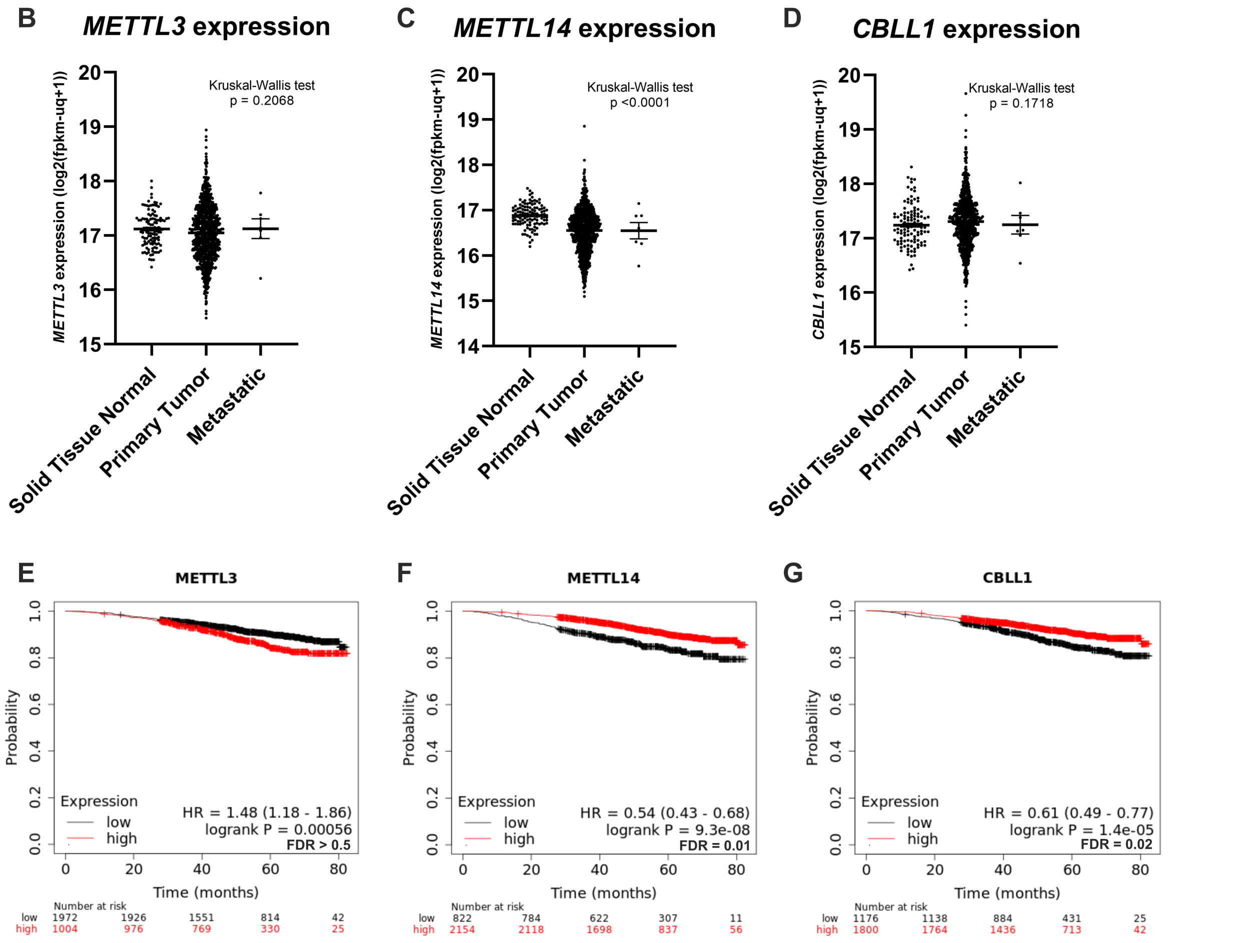

Supplementary Figure 1 Mutations and RNA expression of *METTL3*, *METTL14* and *CBLL1* in breast cancer

(A) Mutations in *METTL3*, *METTL14* and *CBLL1* in the Breast Invasive Carcinoma cohort (TCGA, PanCancer Atlas, n=996). Comparison of tumour and normal breast tissue expression of (B) *METTL3*, (C) *METTL14* and (D) *CBLL1* (TCGA GDC Xena, n=1284). KM plots showing the correlation of (E) *METTL3*, (F) *METTL14* and (G) *CBLL1* expression with overall survival (KM plotter, n=2976).

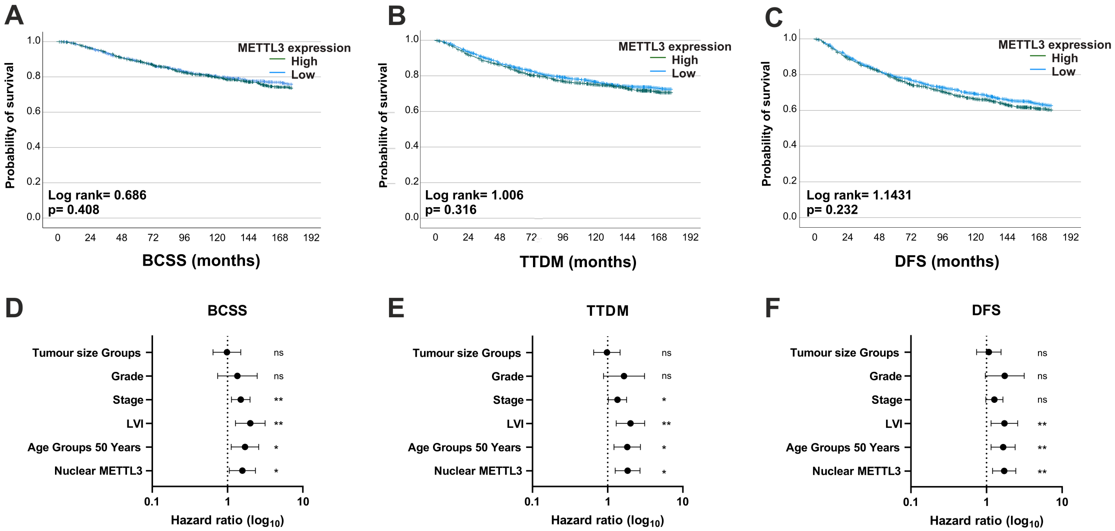

A
B
C
METTL3 expression
High
Low
Probability of survival
Log rank= 0.686
p= 0.408
BCSS (months)
Log rank= 1.006
p= 0.316
TTDM (months)
Log rank= 1.1431
p= 0.232
DFS (months)
D
E
F
BCSS
TTDM
DFS
Tumour size Groups
Grade
Stage
LVI
Age Groups 50 Years
Nuclear METTL3
Hazard ratio (log10)

Supplementary Figure 2 METTL3 expression correlated with survival and prognosis in breast cancer patients

IHC staining of BCa patient samples was assessed by H-score, divided by median into high and low expression groups and correlated with breast cancer-specific survival (BCSS), time to distant metastasis (TTDM) and disease-free survival (DFS). METTL3 staining in all BCa patients, low: ≤105, high: ≥106. (A) Correlation of METTL3 staining and BCSS (n=2115, events at 15 years, low: 262/1210, high: 211/905). (B) Correlation of METTL3 staining and TTDM (n=2115, events at 15 years, low: 304/1210, high: 246/905). (C) Correlation of METTL3 staining and DFS (n=2115, events at 15 years, low: 410/1210, high: 332/905). Correlated using Kaplan Meier estimate and analysed by log-rank test.

Combined multivariate Cox regression analysis of METTL3 nuclear expression, tumour size, tumour grade, tumour stage, LVI and patient age with 15-year (D) BCSS (n=326), (E) TTDM (n=328) and (F) DFS (n=328) in TNBC patient samples. * = $p \leq 0.05$, ** = $p \leq 0.01$, ns = not significant.

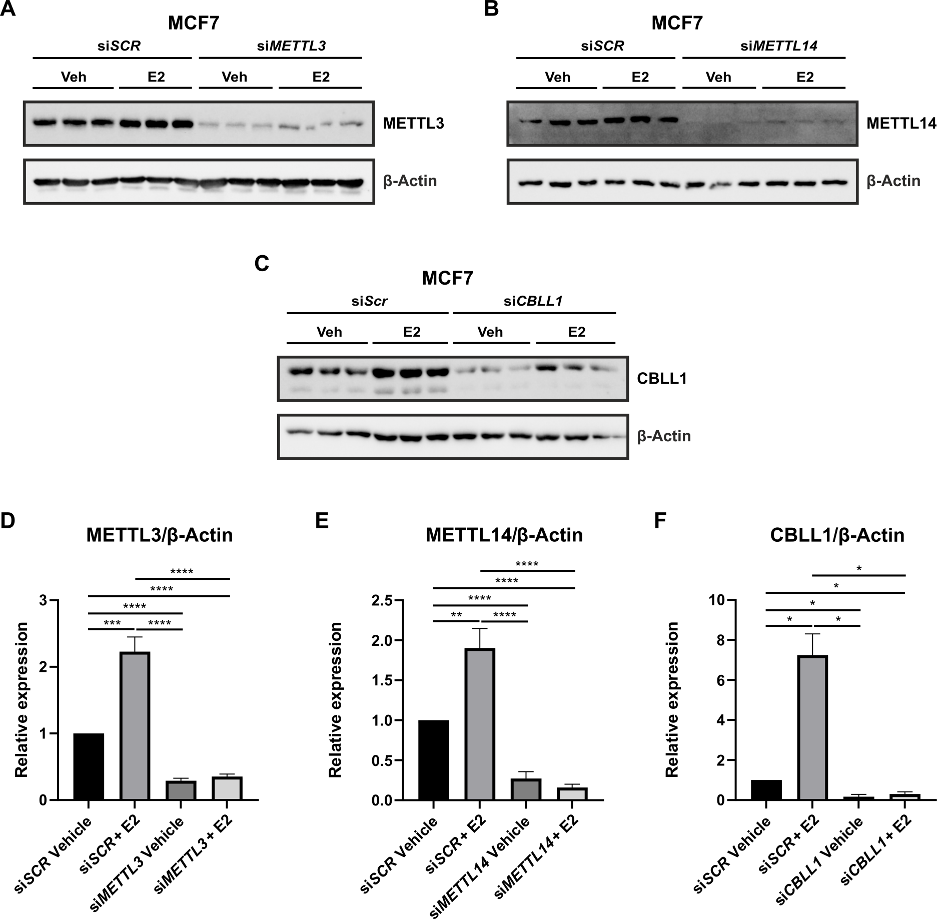
A
MCF7
siSCR
siMETTL3
Veh
E2
Veh
E2
METTL3
β-Actin
B
MCF7
siSCR
siMETTL14
Veh
E2
Veh
E2
METTL14
β-Actin
C
MCF7
siScr
siCBLL1
Veh
E2
Veh
E2
CBLL1
β-Actin
D
METTL3/β-Actin
Relative expression
0
1
2
3
***
****
****
****
****
siSCR Vehicle
siSCR+ E2
siMETTL3 Vehicle
siMETTL3+ E2
E
METTL14/β-Actin
Relative expression
0.0
0.5
1.0
1.5
2.0
2.5
**
****
****
****
****
siSCR Vehicle
siSCR+ E2
siMETTL14 Vehicle
siMETTL14+ E2
F
CBLL1/β-Actin
Relative expression
0
2
4
6
8
10
*
*
*
*
*
siSCR Vehicle
siSCR+ E2
siCBLL1 Vehicle
siCBLL1+ E2

Supplementary Figure 3 Representative images of METTL3, METTL14 and CBLL1 expression. (A) Expression of METTL3 and β-Actin in MCF7 cells treated with siSCR or si*METTL3* and with DMSO vehicle or E2 (n=3); (B) expression of METTL14 and β-Actin in MCF7 cells treated with siSCR or si*METTL14* and with DMSO vehicle or E2 (n=3); (C) expression of CBLL1 and β-Actin in MCF7 cells treated with siSCR or si*CBLL1* and with DMSO vehicle or E2 (n=3). (D) Quantification of METTL3 protein expression in MCF7 cells following METTL3 siRNA-mediated knockdown treated with DMSO vehicle or E2 (n=6); (E) quantification of METTL14 protein expression in MCF7 cells following METTL14 siRNA-mediated knockdown treated with DMSO vehicle or E2 (n=6); (F) quantification of CBLL1 protein expression in MCF7 cells following CBLL1 siRNA-mediated knockdown treated with DMSO vehicle or E2 (n=6). Quantification based on two independent experiments of n=3 (shown in Supplementary Figures 4 & 5). * = $p \leq 0.05$, ** = $p \leq 0.01$, *** = $p \leq 0.001$, **** = $p \leq 0.0001$ by paired ANOVA.

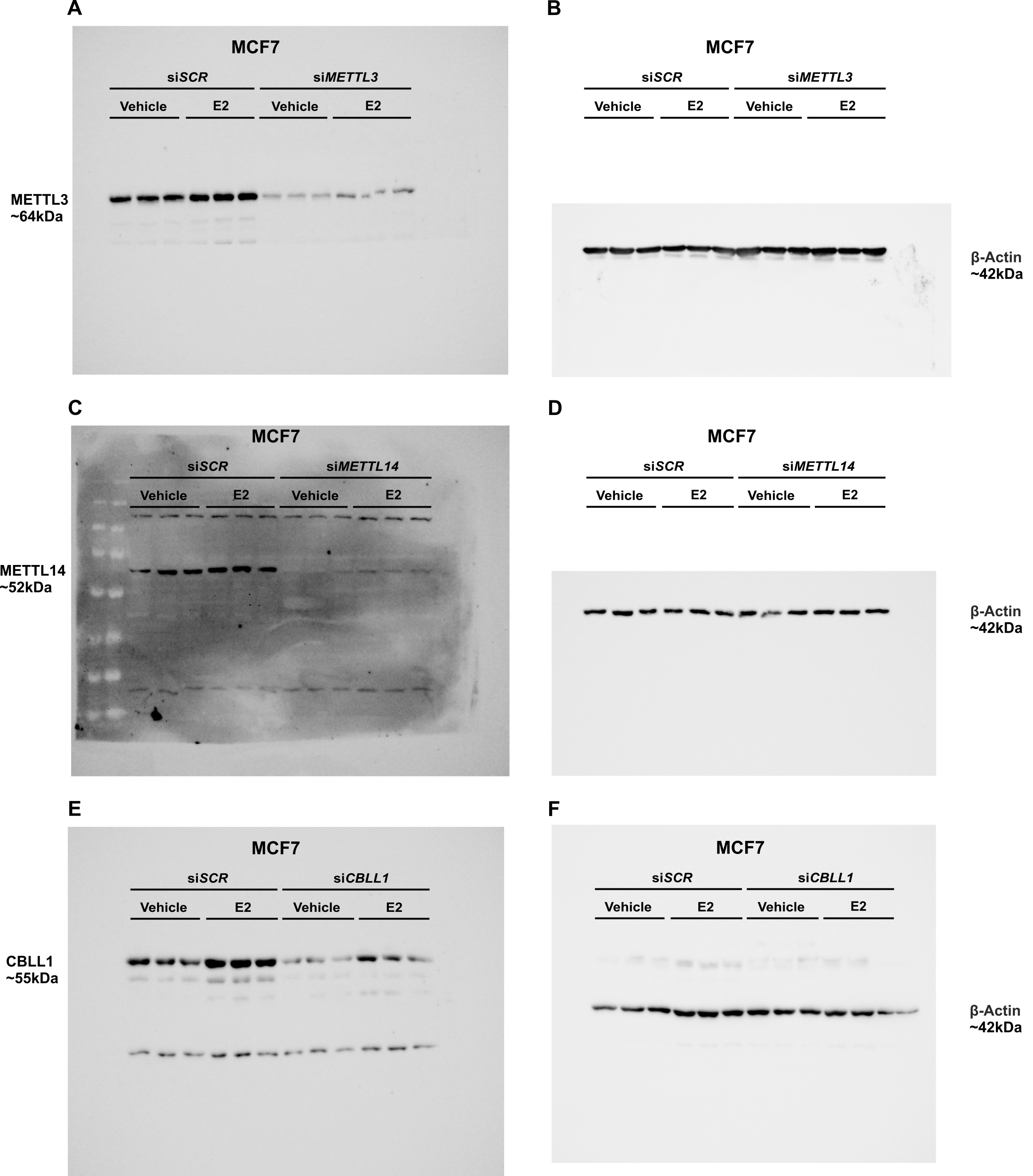
A
MCF7
siSCR
siMETTL3
Vehicle
E2
Vehicle
E2
METTL3
~64kDa
B
MCF7
siSCR
siMETTL3
Vehicle
E2
Vehicle
E2
β-Actin
~42kDa
C
MCF7
siSCR
siMETTL14
Vehicle
E2
Vehicle
E2
METTL14
~52kDa
D
MCF7
siSCR
siMETTL14
Vehicle
E2
Vehicle
E2
β-Actin
~42kDa
E
MCF7
siSCR
siCBLL1
Vehicle
E2
Vehicle
E2
CBLL1
~55kDa
F
MCF7
siSCR
siCBLL1
Vehicle
E2
Vehicle
E2
β-Actin
~42kDa

Supplementary Figure 4. Full uncropped annotated western blot images for first independent experiment (all n=3). Expression of METTL3 (A) and β-Actin (B) in MCF7 cells treated with siSCR or si*METTL3* and with DMSO or E2; expression of METTL14 (C) and β-Actin (D) in MCF7 cells treated with siSCR or si*METTL14* and with DMSO or E2; expression of CBLL1 (E) and β-Actin (F) in MCF7 cells treated with siSCR or si*CBLL1* and with DMSO or E2.

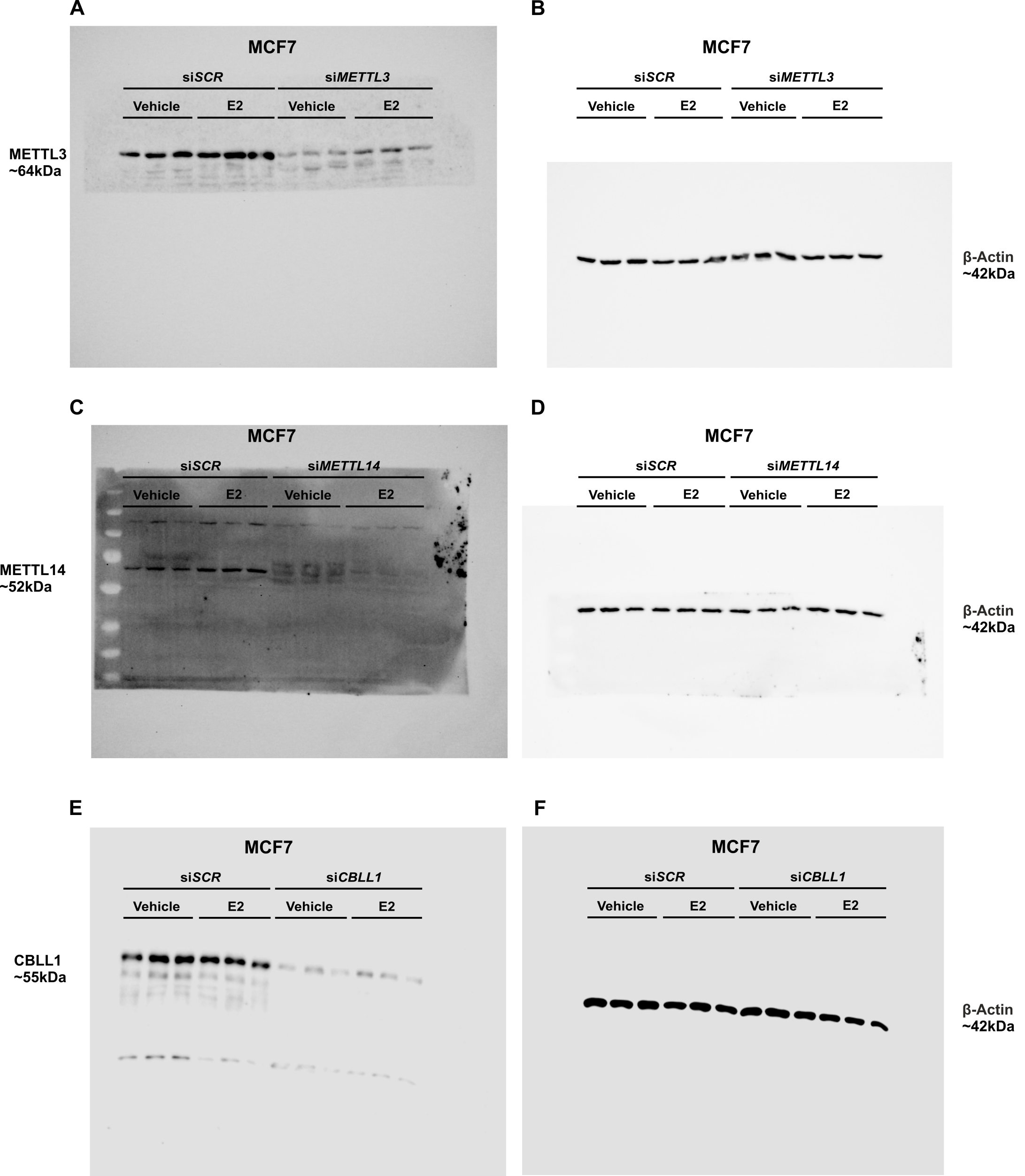
A
MCF7
siSCR
siMETTL3
Vehicle
E2
Vehicle
E2
METTL3
~64kDa
B
MCF7
siSCR
siMETTL3
Vehicle
E2
Vehicle
E2
β-Actin
~42kDa
C
MCF7
siSCR
siMETTL14
Vehicle
E2
Vehicle
E2
METTL14
~52kDa
D
MCF7
siSCR
siMETTL14
Vehicle
E2
Vehicle
E2
β-Actin
~42kDa
E
MCF7
siSCR
siCBLL1
Vehicle
E2
Vehicle
E2
CBLL1
~55kDa
F
MCF7
siSCR
siCBLL1
Vehicle
E2
Vehicle
E2
β-Actin
~42kDa

Supplementary Figure 5. Full uncropped annotated western blot images for second independent experiment (all n=3). Expression of (A) METTL3 and (B) β-Actin in MCF7 cells treated with siSCR or si*METTL3* and with DMSO or E2; expression of (C) METTL14 and (D) β-Actin in MCF7 cells treated with siSCR or si*METTL14* and with DMSO or E2; expression of (E) CBLL1 and (F) β-Actin in MCF7 cells treated with siSCR or si*CBLL1* and with DMSO or E2.

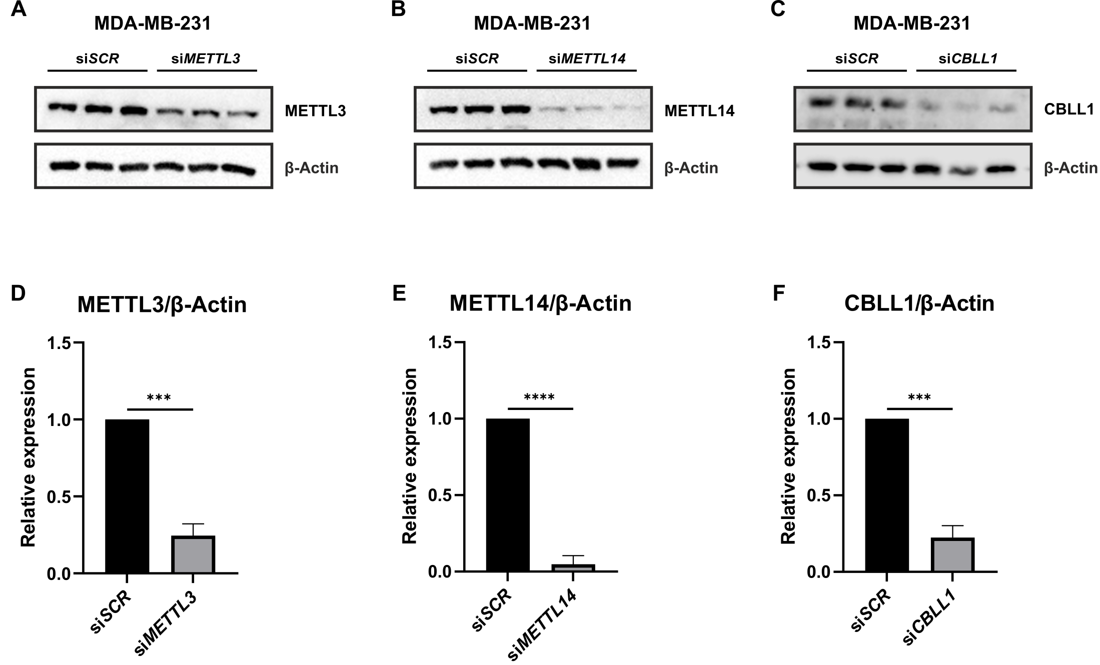
A
MDA-MB-231
siSCR
siMETTL3
METTL3
β-Actin
B
MDA-MB-231
siSCR
siMETTL14
METTL14
β-Actin
C
MDA-MB-231
siSCR
siCBLL1
CBLL1
β-Actin
D
METTL3/β-Actin
Relative expression
1.5
1.0
0.5
0.0
***
siSCR
siMETTL3
E
METTL14/β-Actin
Relative expression
1.5
1.0
0.5
0.0
****
siSCR
siMETTL14
F
CBLL1/β-Actin
Relative expression
1.5
1.0
0.5
0.0
***
siSCR
siCBLL1

Supplementary Figure 6. Representative images of METTL3, METTL14 and CBLL1 expression. (A) Expression of METTL3 and β-Actin in MDA-MB-231 treated with siSCR or si*METTL3* (n=3); (B) expression of METTL14 and β-Actin in MDA-MB-231 treated with siSCR or si*METTL14* (n=3); (C) expression of CBLL1 and β-Actin in MDA-MB-231 treated with siSCR or si*CBLL1* (n=3). (D) Quantification of METTL3 protein expression in MDA-MB-231 following METTL3 siRNA-mediated knockdown (n=6); (E) quantification of METTL14 protein expression in MDA-MB-231 following METTL14 siRNA-mediated knockdown (n=6); (F) quantification of CBLL1 protein expression in MDA-MB-231 following CBLL1 siRNA-mediated knockdown (n=6). Quantification based on two independent experiments of n=3 (shown in Supplementary Figures 7 and 8). * = $p \leq 0.05$, ** = $p \leq 0.01$, *** = $p \leq 0.001$, **** = $p \leq 0.0001$ by paired t-test.

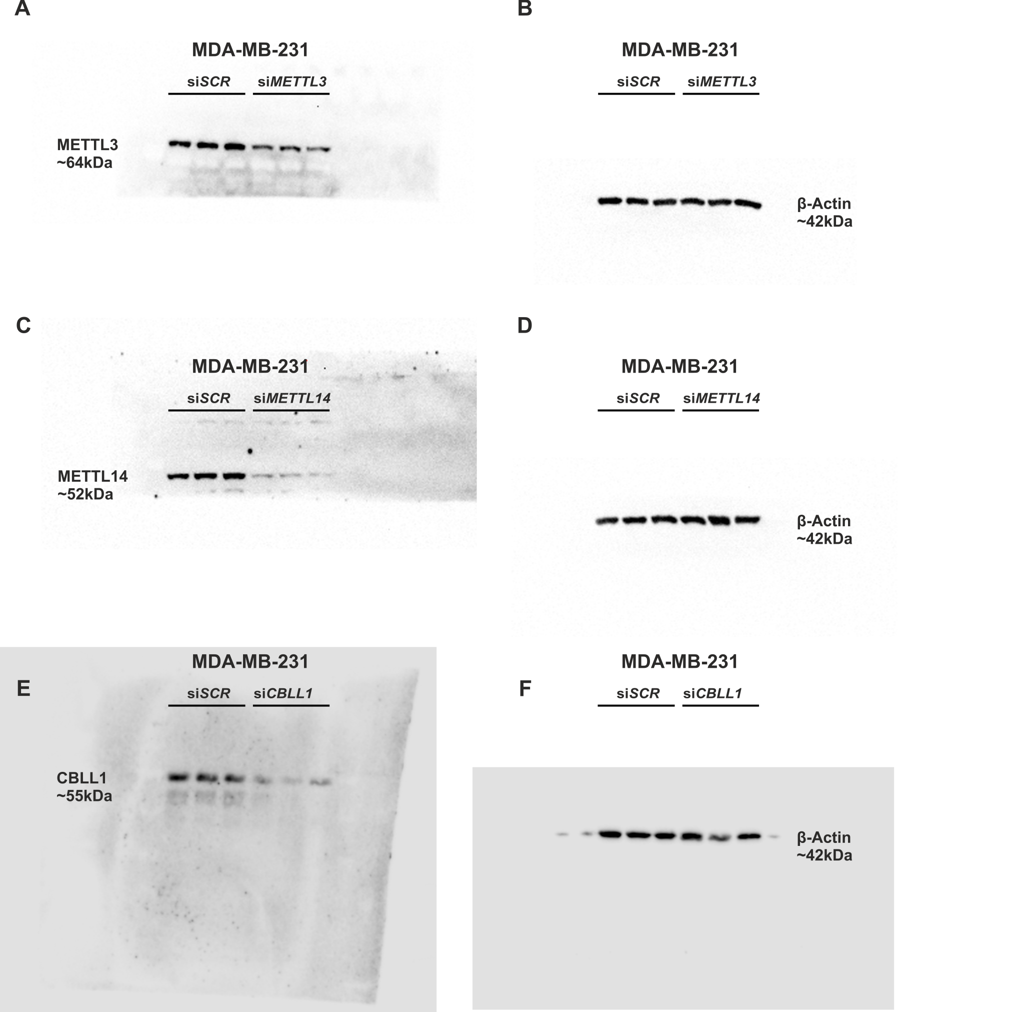
A
MDA-MB-231
siSCR
siMETTL3
METTL3 ~64kDa
B
MDA-MB-231
siSCR
siMETTL3
β-Actin ~42kDa
C
MDA-MB-231
siSCR
siMETTL14
METTL14 ~52kDa
D
MDA-MB-231
siSCR
siMETTL14
β-Actin ~42kDa
E
MDA-MB-231
siSCR
siCBLL1
CBLL1 ~55kDa
F
MDA-MB-231
siSCR
siCBLL1
β-Actin ~42kDa

Supplementary Figure 7. Full uncropped annotated western blot images for first independent experiment (all n=3). Expression of (A) METTL3 and (B) β-Actin in MDA-MB-231 treated with siSCR or si*METTL3*; expression of (C) METTL14 and (D) β-Actin in MDA-MB-231 treated with siSCR or si*METTL14*; expression of (E) CBLL1 and (F) β-Actin in MDA-MB-231 treated with siSCR or si*CBLL1*.

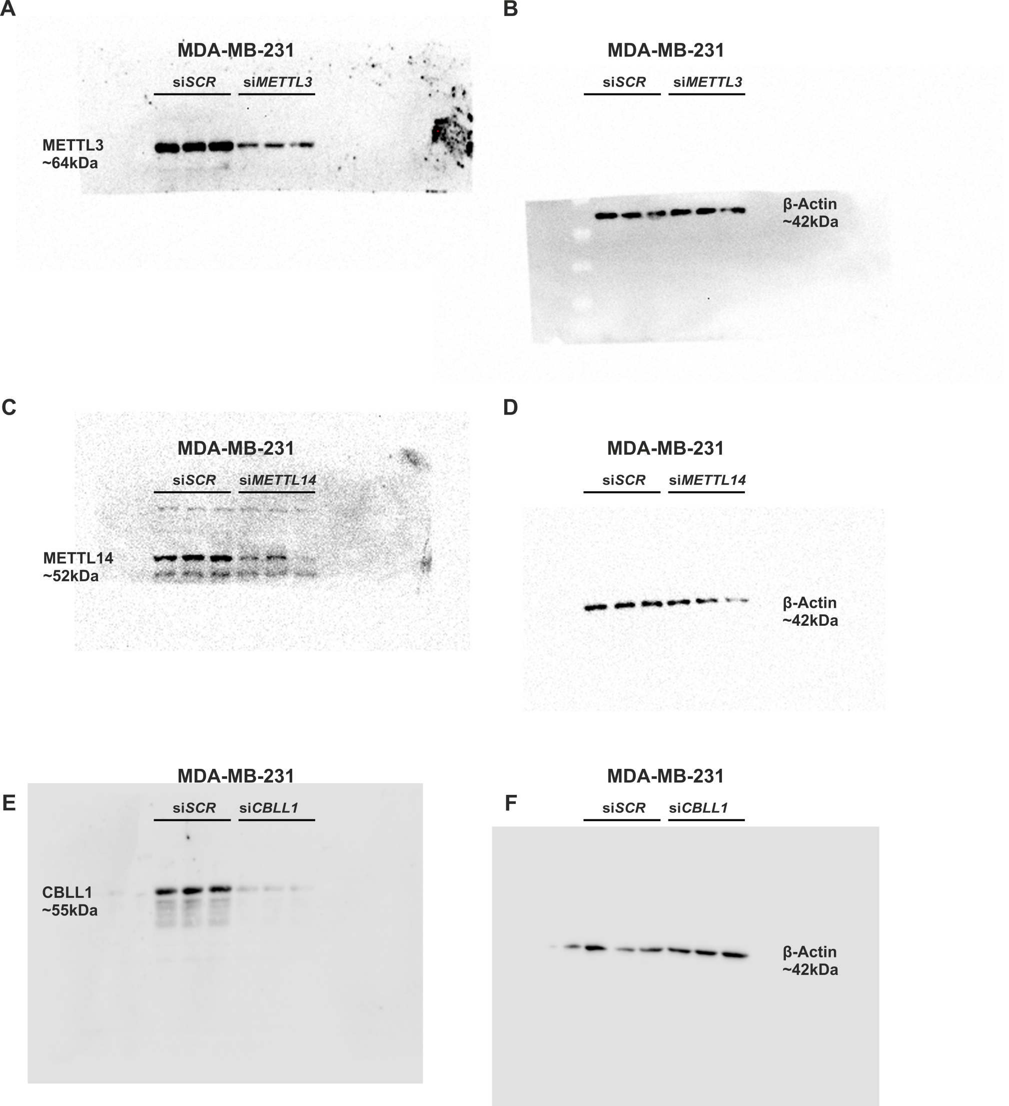
A
MDA-MB-231
siSCR
siMETTL3
METTL3
~64kDa
B
MDA-MB-231
siSCR
siMETTL3
β-Actin
~42kDa
C
MDA-MB-231
siSCR
siMETTL14
METTL14
~52kDa
D
MDA-MB-231
siSCR
siMETTL14
β-Actin
~42kDa
E
MDA-MB-231
siSCR
siCBLL1
CBLL1
~55kDa
F
MDA-MB-231
siSCR
siCBLL1
β-Actin
~42kDa

Supplementary Figure 8. Full uncropped annotated western blot images for second independent experiment (all n=3). Expression of (A) METTL3 and (B) β-Actin in MDA-MB-231 treated with siSCR or si*METTL3*; expression of (C) METTL14 and (D) β-Actin in MDA-MB-231 treated with siSCR or si*METTL14*; expression of (E) CBLL1 and (F) β-Actin in MDA-MB-231 treated with siSCR or si*CBLL1*.

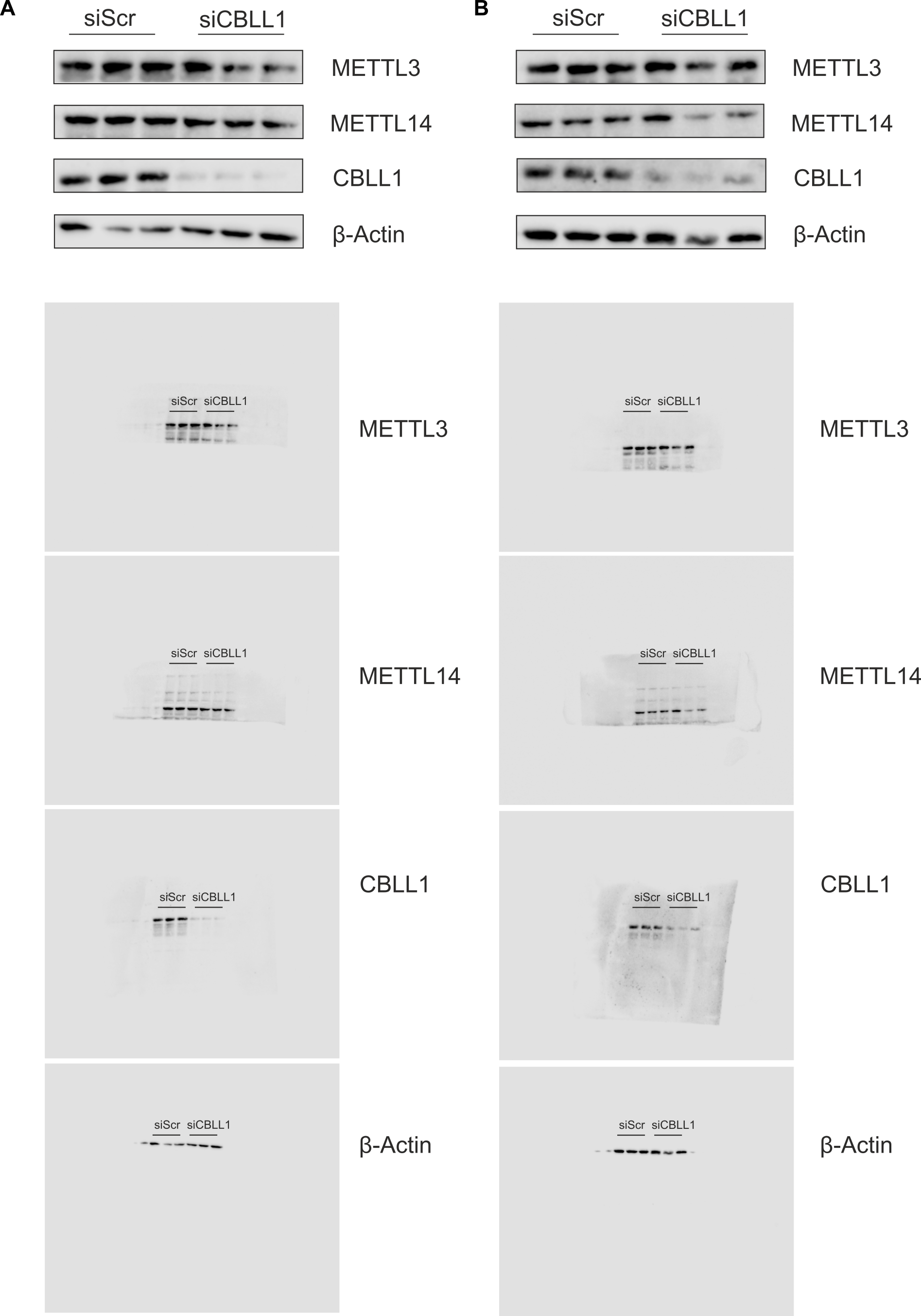
A
B
siScr
siCBLL1
METTL3
METTL14
CBLL1
β-Actin

Supplementary Figure 9. Annotated western blots for the expression of METTL3, METTL14, CBLL1 and β-Actin in siRNA-mediated CBLL1 knockdown in MDA-MB-231 with full uncropped annotated western blot images below. (A) First independent experiment and (B) second independent experiment.

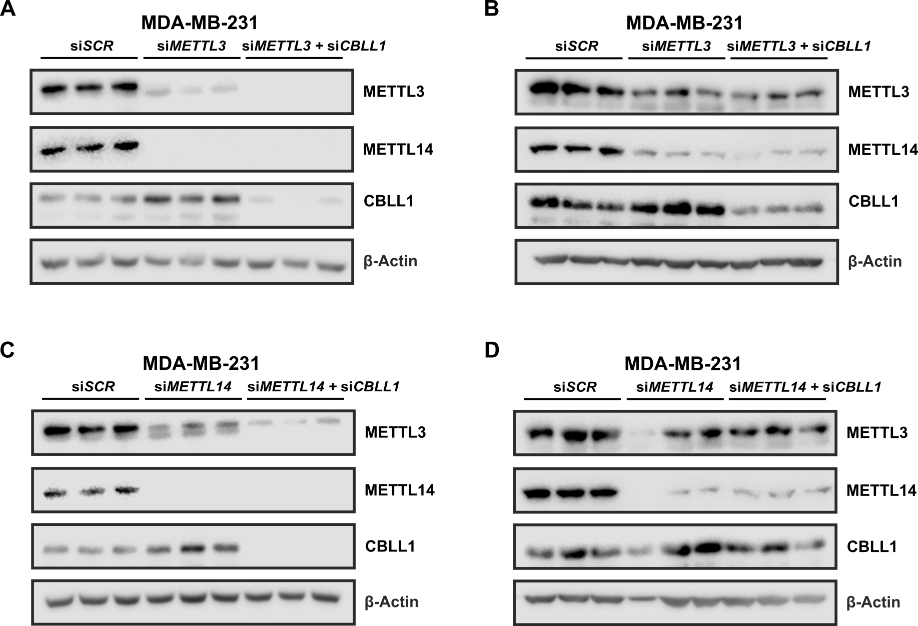
A
MDA-MB-231
siSCR
siMETTL3
siMETTL3 + siCBLL1
METTL3
METTL14
CBLL1
β-Actin
B
MDA-MB-231
siSCR
siMETTL3
siMETTL3 + siCBLL1
METTL3
METTL14
CBLL1
β-Actin
C
MDA-MB-231
siSCR
siMETTL14
siMETTL14 + siCBLL1
METTL3
METTL14
CBLL1
β-Actin
D
MDA-MB-231
siSCR
siMETTL14
siMETTL14 + siCBLL1
METTL3
METTL14
CBLL1
β-Actin

Supplementary Figure 10. Expression of METTL3, METTL14, CBLL1 and β-Actin in MDA-MB-231 treated with either siSCR, si*METTL3*, or a combination of si*METTL3* and si*CBLL1* (n=3) (A) experiment 1 and (B) experiment 2; expression of METTL3, METTL14, CBLL1 and β-actin in MDA-MB-231 treated with either siSCR, si*METTL14*, or a combination of si*METTL14* and si*CBLL1* (n=3) (C) experiment 1 and (D) experiment 2. Full uncropped western blot images shown in Supplementary Figure 11 and 12.

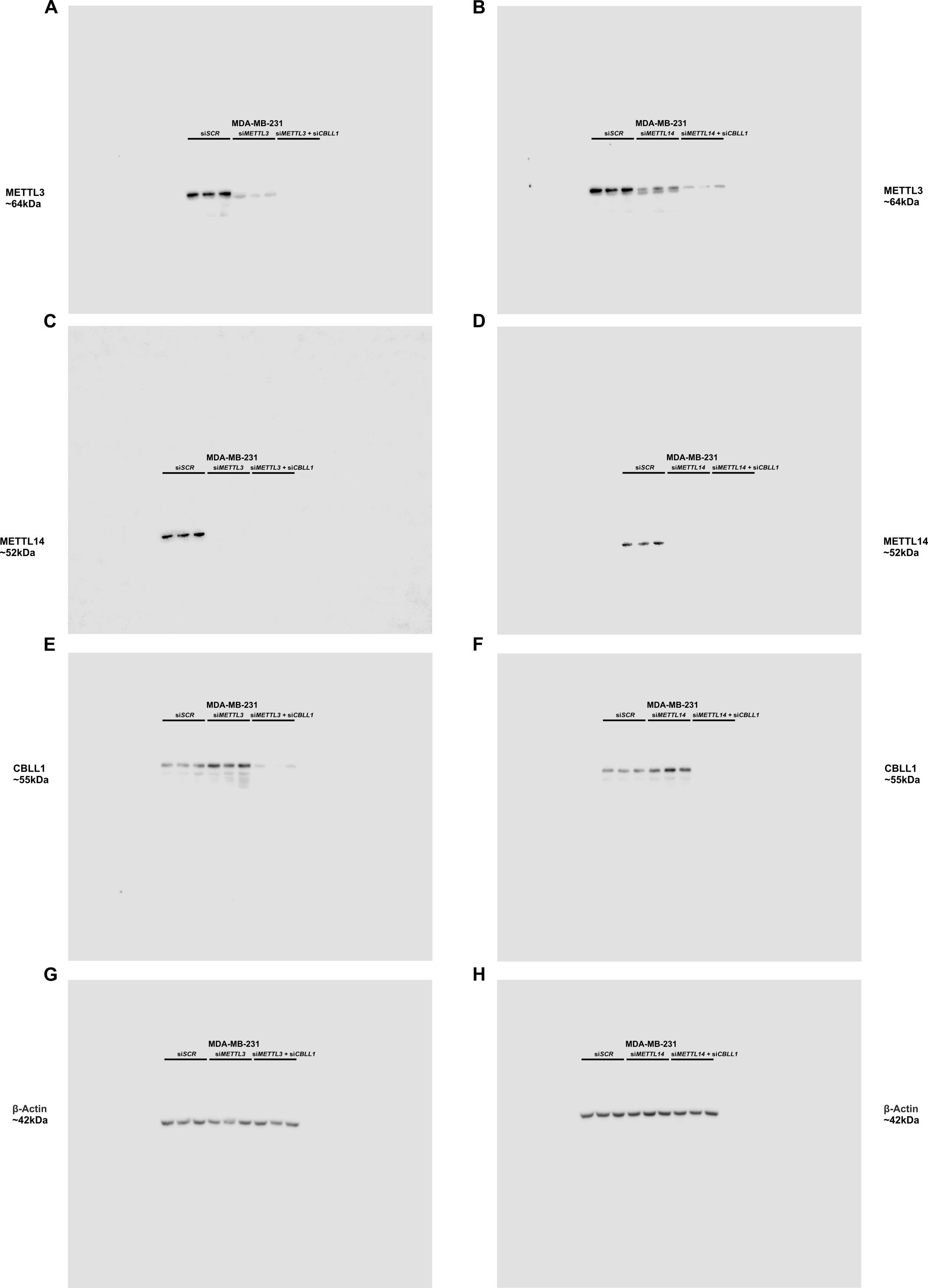
A
MDA-MB-231
siSCR
siMETTL3
siMETTL3 + siCBLL1
METTL3
~64kDa
B
MDA-MB-231
siSCR
siMETTL14
siMETTL14 + siCBLL1
METTL3
~64kDa
C
MDA-MB-231
siSCR
siMETTL3
siMETTL3 + siCBLL1
METTL14
~52kDa
D
MDA-MB-231
siSCR
siMETTL14
siMETTL14 + siCBLL1
METTL14
~52kDa
E
MDA-MB-231
siSCR
siMETTL3
siMETTL3 + siCBLL1
CBLL1
~55kDa
F
MDA-MB-231
siSCR
siMETTL14
siMETTL14 + siCBLL1
CBLL1
~55kDa
G
MDA-MB-231
siSCR
siMETTL3
siMETTL3 + siCBLL1
β-Actin
~42kDa
H
MDA-MB-231
siSCR
siMETTL14
siMETTL14 + siCBLL1
β-Actin
~42kDa

Supplementary Figure 11. Full uncropped annotated western blot images for first independent experiment (all n=3). Expression of (A) METTL3, (C) METTL14, (E) CBLL1 and (G) β-Actin in MDA-MB-231 treated with either siSCR, si*METTL3*, or a combination of si*METTL3* and si*CBLL1* (n=3). Expression of (B) METTL3, (D) METTL14, (F) CBLL1 and (H) β-Actin in MDA-MB-231 treated with either siSCR, si*METTL14*, or a combination of si*METTL14* and si*CBLL1* (n=3).

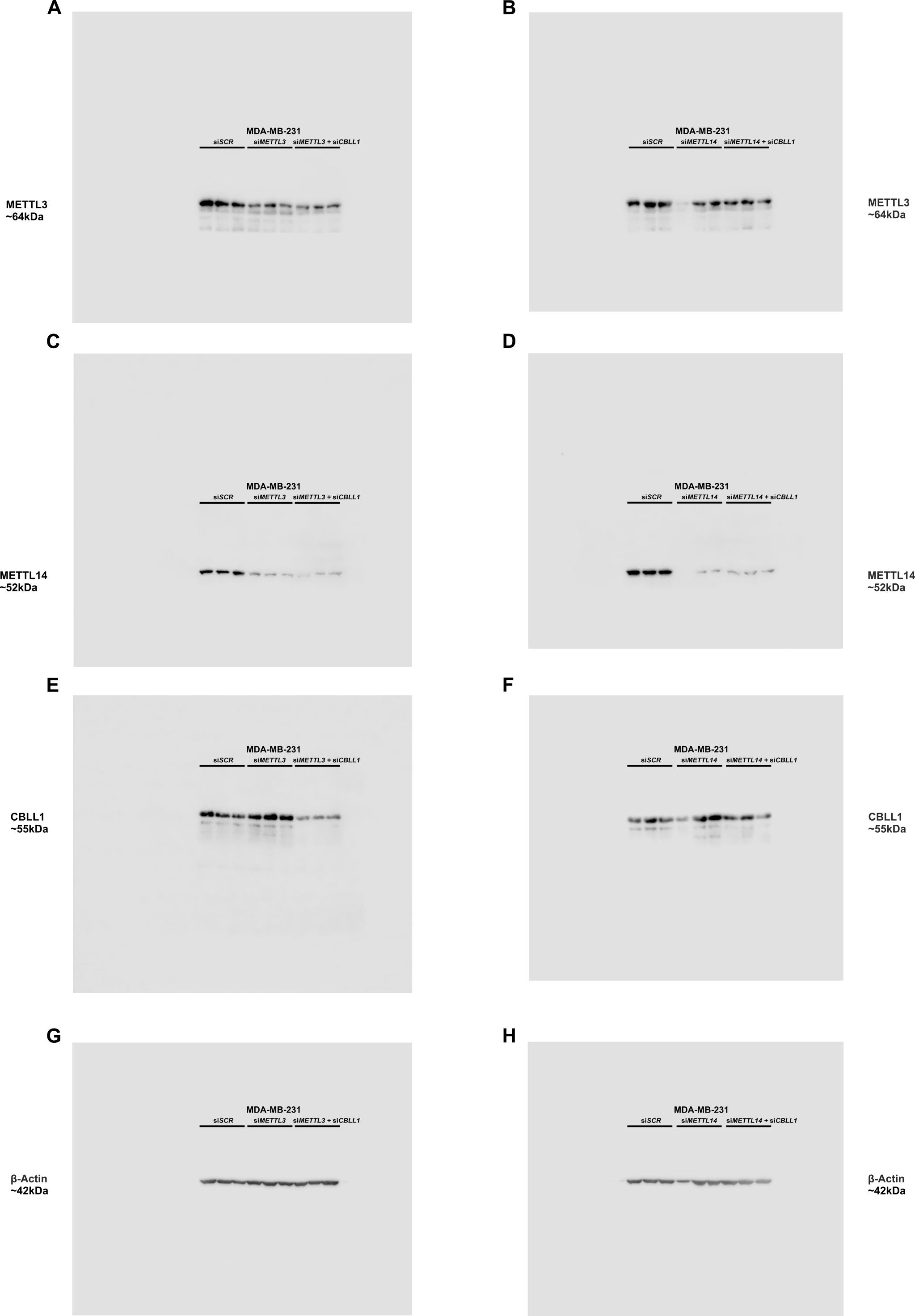
A
MDA-MB-231
siSCR
siMETTL3
siMETTL3 + siCBLL1
METTL3
~64kDa
B
MDA-MB-231
siSCR
siMETTL14
siMETTL14 + siCBLL1
METTL3
~64kDa
C
MDA-MB-231
siSCR
siMETTL3
siMETTL3 + siCBLL1
METTL14
~52kDa
D
MDA-MB-231
siSCR
siMETTL14
siMETTL14 + siCBLL1
METTL14
~52kDa
E
MDA-MB-231
siSCR
siMETTL3
siMETTL3 + siCBLL1
CBLL1
~55kDa
F
MDA-MB-231
siSCR
siMETTL14
siMETTL14 + siCBLL1
CBLL1
~55kDa
G
MDA-MB-231
siSCR
siMETTL3
siMETTL3 + siCBLL1
β-Actin
~42kDa
H
MDA-MB-231
siSCR
siMETTL14
siMETTL14 + siCBLL1
β-Actin
~42kDa

Supplementary Figure 12. Full uncropped annotated western blot images for second independent experiment (all n=3). Expression of (A) METTL3, (C) METTL14, (E) CBLL1 and (G) β-Actin in MDA-MB-231 treated with either siSCR, si*METTL3*, or a combination of si*METTL3* and si*CBLL1* (n=3). Expression of (B) METTL3, (D) METTL14, (F) CBLL1 and (H) β-Actin in MDA-MB-231 treated with either siSCR, si*METTL14*, or a combination of si*METTL14* and si*CBLL1* (n=3).

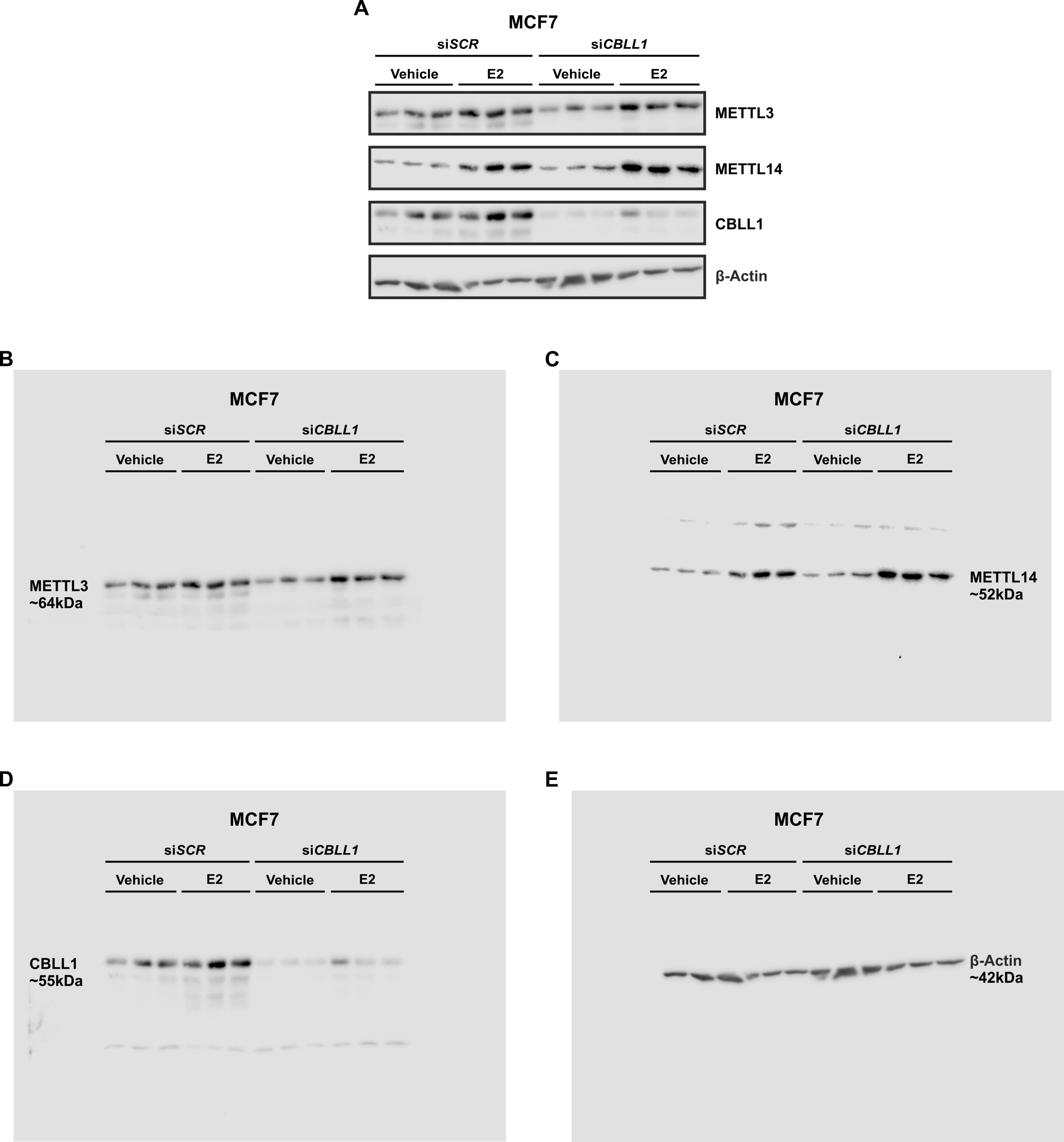
A
MCF7
siSCR
siCBLL1
Vehicle
E2
Vehicle
E2
METTL3
METTL14
CBLL1
β-Actin
B
MCF7
siSCR
siCBLL1
Vehicle
E2
Vehicle
E2
METTL3
~64kDa
C
MCF7
siSCR
siCBLL1
Vehicle
E2
Vehicle
E2
METTL14
~52kDa
D
MCF7
siSCR
siCBLL1
Vehicle
E2
Vehicle
E2
CBLL1
~55kDa
E
MCF7
siSCR
siCBLL1
Vehicle
E2
Vehicle
E2
β-Actin
~42kDa

Supplementary Figure 13. (A) Expression of METTL3, METTL14, CBLL1 and β-Actin in MCF7 treated with siSCR or si*CBLL1* and with DMSO or E2 (n=3). Full uncropped annotated western blot images for first independent experiment (all n=3); expression of (B) METTL3, (C) METTL14, (D) CBLL1 and (E) β-Actin in MCF7 cells treated with siSCR or si*CBLL1* and with DMSO or E2.

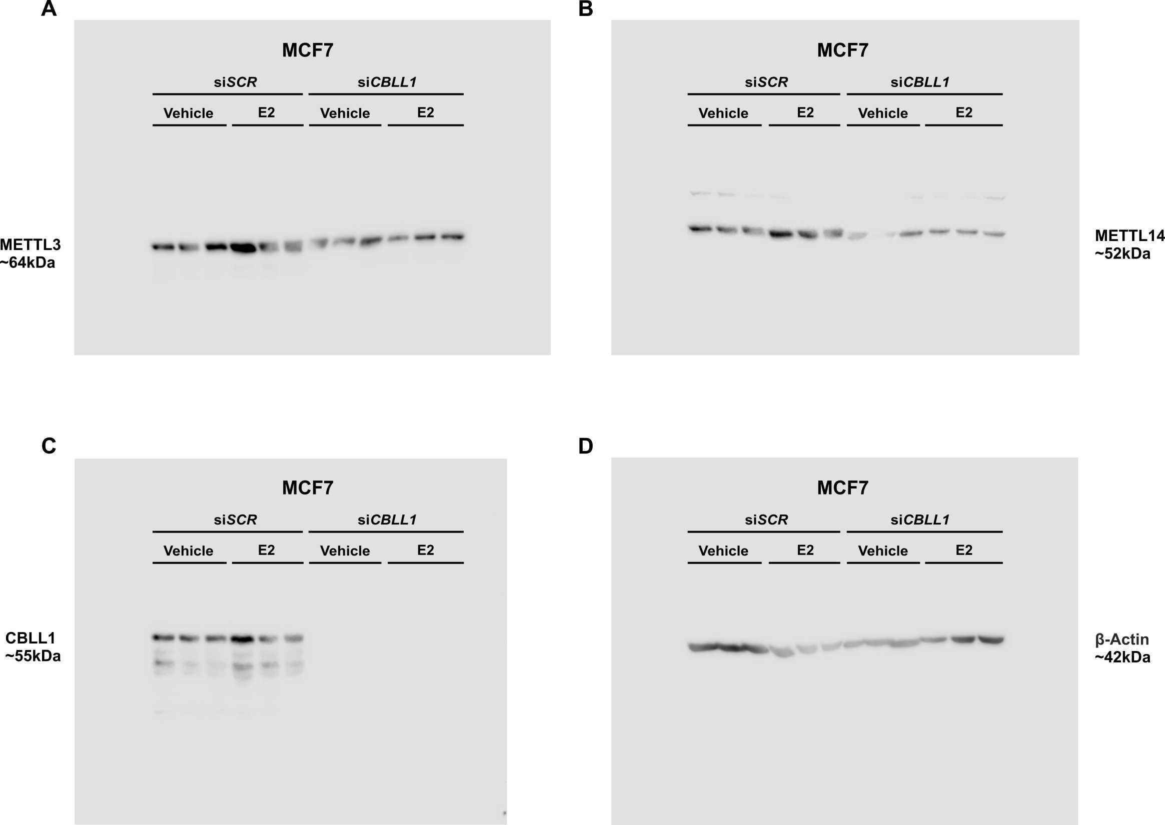
A
MCF7
siSCR
siCBLL1
Vehicle
E2
Vehicle
E2
METTL3
~64kDa
B
MCF7
siSCR
siCBLL1
Vehicle
E2
Vehicle
E2
METTL14
~52kDa
C
MCF7
siSCR
siCBLL1
Vehicle
E2
Vehicle
E2
CBLL1
~55kDa
D
MCF7
siSCR
siCBLL1
Vehicle
E2
Vehicle
E2
β-Actin
~42kDa

Supplementary Figure 14. Full uncropped annotated western blot images for second independent experiment (all n=3); expression of (A) METTL3, (B) METTL14, (C) CBLL1 and (D) β-Actin in MCF7 treated with siSCR or si*CBLL1* and with DMSO or E2.

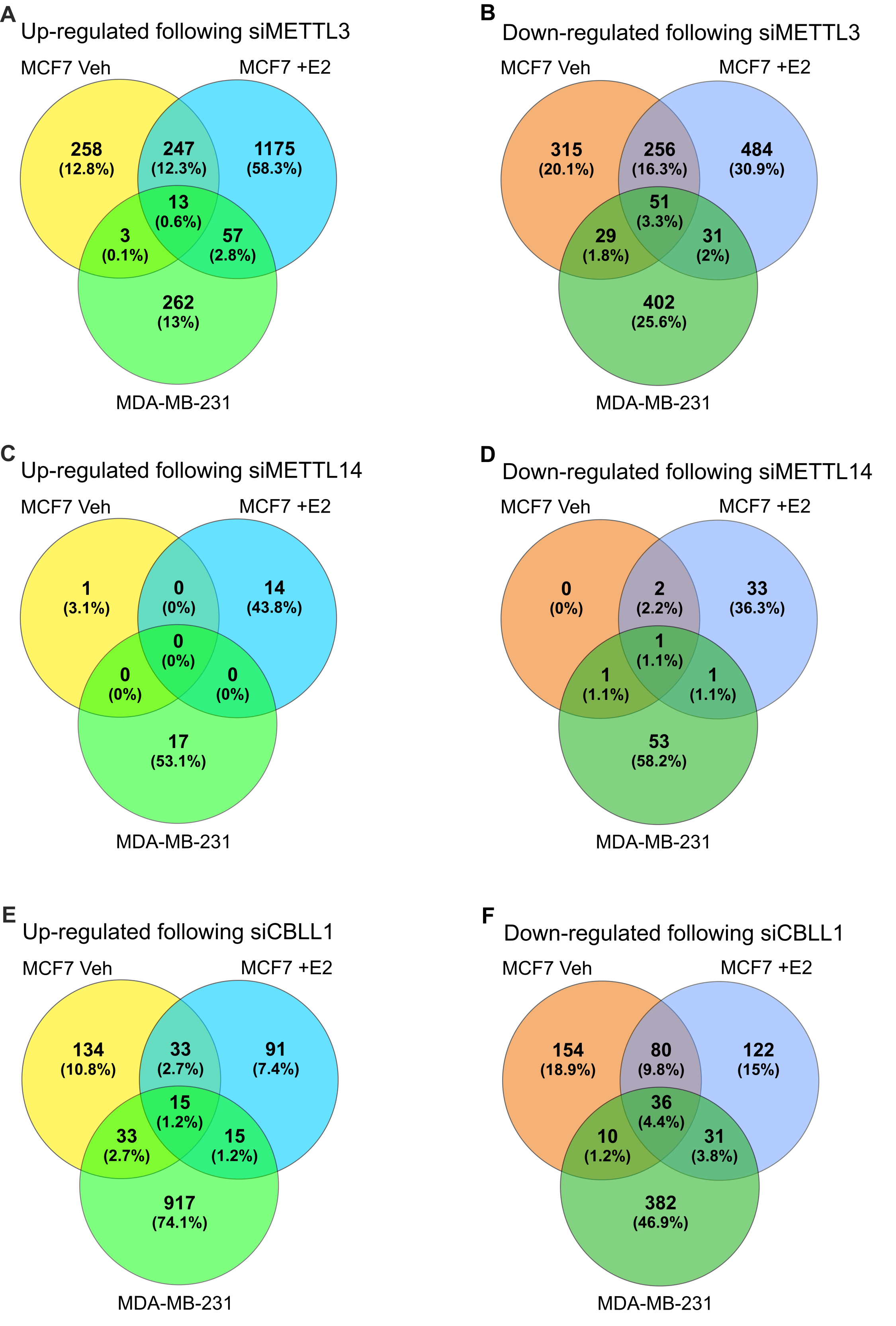
A
Up-regulated following siMETTL3
MCF7 Veh
MCF7 +E2
258 (12.8%)
247 (12.3%)
1175 (58.3%)
13 (0.6%)
3 (0.1%)
57 (2.8%)
262 (13%)
MDA-MB-231
B
Down-regulated following siMETTL3
MCF7 Veh
MCF7 +E2
315 (20.1%)
256 (16.3%)
484 (30.9%)
51 (3.3%)
29 (1.8%)
31 (2%)
402 (25.6%)
MDA-MB-231
C
Up-regulated following siMETTL14
MCF7 Veh
MCF7 +E2
1 (3.1%)
0 (0%)
14 (43.8%)
0 (0%)
0 (0%)
0 (0%)
17 (53.1%)
MDA-MB-231
D
Down-regulated following siMETTL14
MCF7 Veh
MCF7 +E2
0 (0%)
2 (2.2%)
33 (36.3%)
1 (1.1%)
1 (1.1%)
1 (1.1%)
53 (58.2%)
MDA-MB-231
E
Up-regulated following siCBLL1
MCF7 Veh
MCF7 +E2
134 (10.8%)
33 (2.7%)
91 (7.4%)
15 (1.2%)
33 (2.7%)
15 (1.2%)
917 (74.1%)
MDA-MB-231
F
Down-regulated following siCBLL1
MCF7 Veh
MCF7 +E2
154 (18.9%)
80 (9.8%)
122 (15%)
36 (4.4%)
10 (1.2%)
31 (3.8%)
382 (46.9%)
MDA-MB-231

Supplementary Figure 15 Summary of differentially expressed genes in breast cancer cell lines in response to siRNA-mediated METTL3, METTL14 and CBLL1 knockdown

Summary of overlap in DEGs in MCF7 +/-E2, and MDA-MB-231. (A) Upregulated expression following METTL3 depletion, (B) downregulated expression following METTL3 depletion, (C) upregulated expression following METTL14 depletion, (D) downregulated expression following METTL14 depletion, (E) upregulated expression following CBLL1 depletion, (F) downregulated expression following CBLL1 depletion. Significant differential gene expression= FC ± 1.5 and adjusted p-value < 0.05.

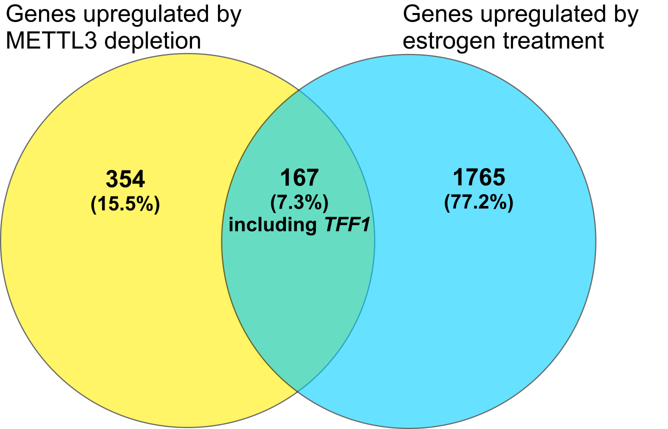
Genes upregulated by METTL3 depletion
Genes upregulated by estrogen treatment
354
(15.5%)
167
(7.3%)
including *TFF1*
1765
(77.2%)

Supplementary Figure 16 METTL3 regulation of estrogen-induced genes in MCF7

Venn diagram of genes significantly upregulated by METTL3 depletion (increased in si*METTL3* compared to siSCR) compared with genes significantly up regulated by estrogen (increased in siSCR +E2 compared to siSCR Vehicle). Significant differential gene expression= FC ± 1.5 and adjusted p-value < 0.05.

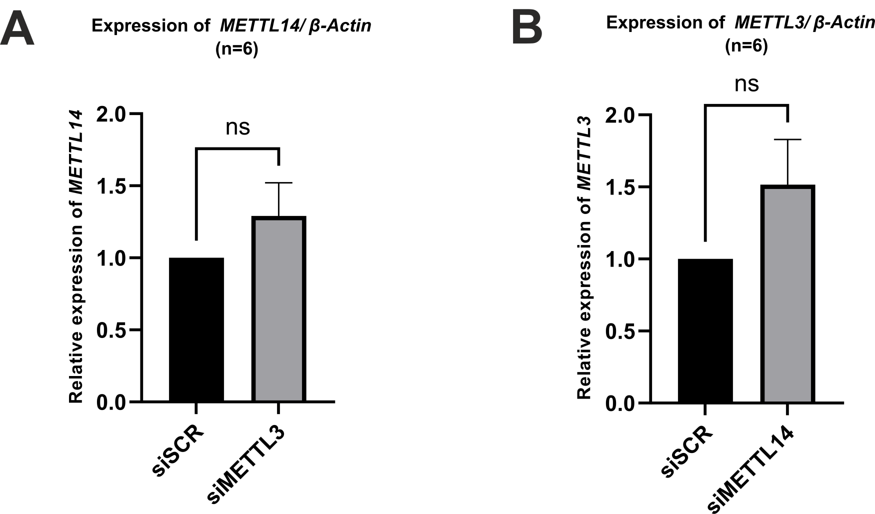
A
Expression of METTL14/ β-Actin
(n=6)
Relative expression of METTL14
2.0
1.5
1.0
0.5
0.0
ns
siSCR
siMETTL3
B
Expression of METTL3/ β-Actin
(n=6)
Relative expression of METTL3
2.0
1.5
1.0
0.5
0.0
ns
siSCR
siMETTL14

Supplementary Figure 17 Depletion of one catalytic heterodimer protein does not alter the transcript expression of the other MTC protein in MDA-MB-231

qRT-PCR of *METTL3* and *METTL14* expression following siRNA-mediated depletion of METTL3 and METTL14. (A) *METTL14* expression following METTL3 depletion and (B) *METTL3* expression following METTL14 depletion in MDA-MB-231. Expression relative to *β-Actin*. * = $p < 0.05$, ** = $p < 0.005$, *** = $p < 0.001$, **** = $p < 0.0001$, ns = not significant.

A

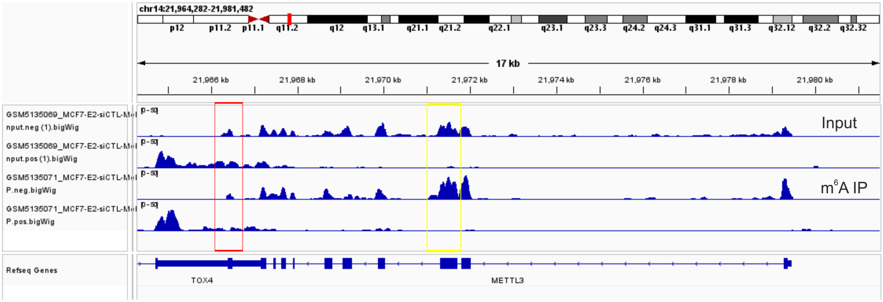

chr14:21,964,282-21,981,482
17 kb
21,966 kb
21,968 kb
21,970 kb
21,972 kb
21,974 kb
21,976 kb
21,978 kb
21,980 kb
GSM5135069_MCF7-E2-siCTL-MeInput.neg (1).bigWig
GSM5135069_MCF7-E2-siCTL-MeInput.pos (1).bigWig
GSM5135071_MCF7-E2-siCTL-MeIP.neg.bigWig
GSM5135071_MCF7-E2-siCTL-MeIP.pos.bigWig
Input
m6A IP
Refseq Genes
TOX4
METTL3

B

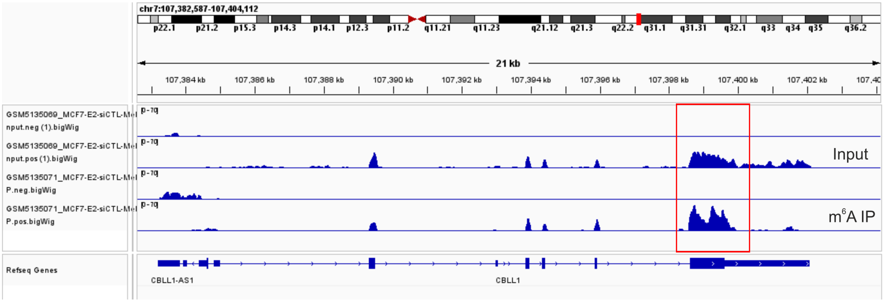

chr7:107,382,587-107,404,112
21 kb
107,384 kb
107,386 kb
107,388 kb
107,390 kb
107,392 kb
107,394 kb
107,396 kb
107,398 kb
107,400 kb
107,402 kb
GSM5135069_MCF7-E2-siCTL-MeInput.neg (1).bigWig
GSM5135069_MCF7-E2-siCTL-MeInput.pos (1).bigWig
GSM5135071_MCF7-E2-siCTL-MeIP.neg.bigWig
GSM5135071_MCF7-E2-siCTL-MeIP.pos.bigWig
Input
m6A IP
Refseq Genes
CBLL1-AS1
CBLL1

C

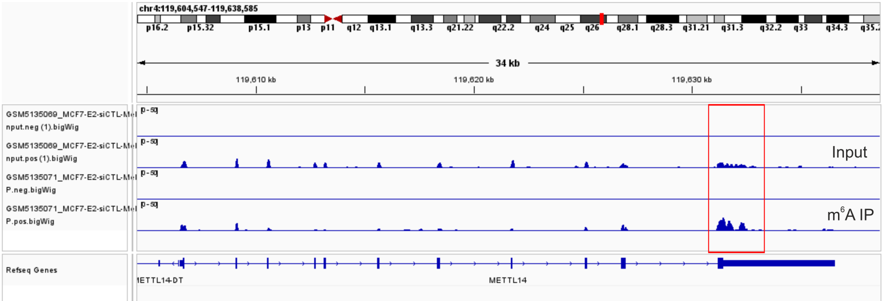

chr4:119,604,547-119,638,585
34 kb
119,610 kb
119,620 kb
119,630 kb
GSM5135069_MCF7-E2-siCTL-MeInput.neg (1).bigWig
GSM5135069_MCF7-E2-siCTL-MeInput.pos (1).bigWig
GSM5135071_MCF7-E2-siCTL-MeIP.neg.bigWig
GSM5135071_MCF7-E2-siCTL-MeIP.pos.bigWig
Input
m6A IP
Refseq Genes
METTL14-DT
METTL14

Supplementary Figure 18 Regulation of the m^{6}A methylation complex components by m^{6}A methylation.

Evidence of the regulation of (A) *METTL3*, (B) *CBLL1* and (C) *METTL14* by m^{6}A methylation in MCF7, MeRIP-Seq data obtained from GEO series GSE143441. Peaks in second panel (m^{6}A enriched) compared to first panel (no enrichment control) show area of m^{6}A enrichment within gene. Red box highlights the last exon of each gene and a yellow box highlights the third exon of *METTL3*.

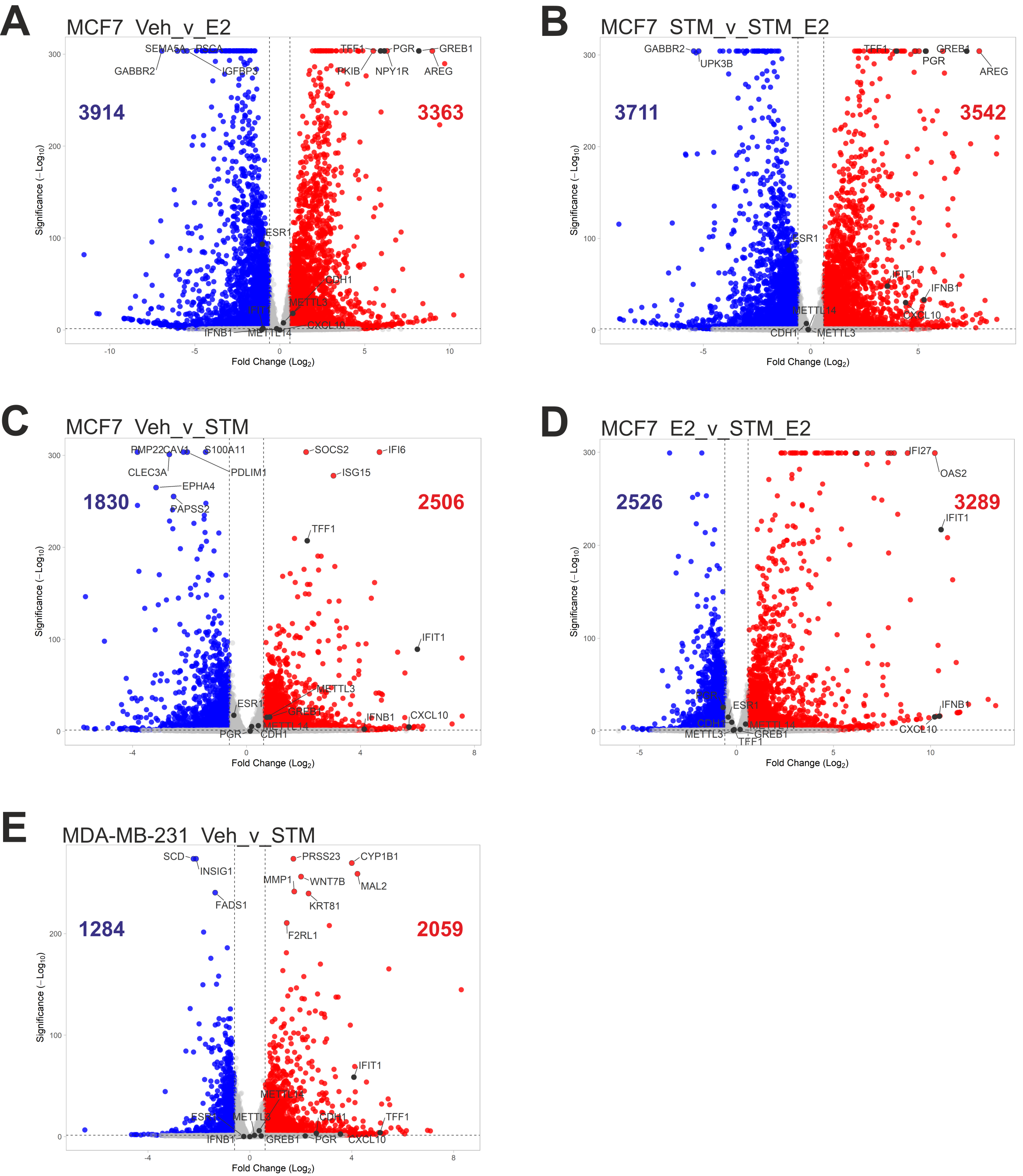
A
MCF7 Veh_v_E2
3914
3363
Significance (−Log10)
Fold Change (Log2)
SEMA5A
PSCA
GABBR2
IGFBP3
TFF1
PGR
GREB1
PKIB
NPY1R
AREG
ESR1
CDH1
METTL3
IFIT1
CXCL10
IFNB1
METTL14
B
MCF7 STM_v_STM_E2
3711
3542
GABBR2
UPK3B
TFF1
GREB1
PGR
AREG
ESR1
IFIT1
IFNB1
METTL14
CXCL10
CDH1
METTL3
C
MCF7 Veh_v_STM
1830
2506
PMP22
CAV1
S100A11
SOCS2
IFI6
CLEC3A
PDLIM1
ISG15
EPHA4
PAPSS2
TFF1
IFIT1
METTL3
ESR1
GREB1
IFNB1
CXCL10
METTL14
PGR
CDH1
D
MCF7 E2_v_STM_E2
2526
3289
IFI27
OAS2
IFIT1
PGR
ESR1
IFNB1
CDH1
METTL14
METTL3
GREB1
TFF1
CXCL10
E
MDA-MB-231 Veh_v_STM
1284
2059
SCD
INSIG1
PRSS23
CYP1B1
MMP1
WNT7B
MAL2
KRT81
FADS1
F2RL1
IFIT1
METTL14
ESR1
METTL3
CDH1
TFF1
IFNB1
GREB1
PGR
CXCL10

Supplementary Figure 19 Differential gene expression with METTL3 inhibition in cell lines MCF7 and MDA-MB-231

RNA-seq reveals significantly differentially expressed genes with METTL3 inhibition. Volcano plot representing the Log2 fold change of differentially expressed genes upon estrogen treatment and METTL3 inhibition in cell line MCF7; Veh vs E2 (A), STM2457 vs STM2457 E2 (B), Veh vs STM2457 (C), and E2 vs STM2457 E2 (D). Volcano plot representing the Log2 fold change of differentially expressed genes upon METTL3 inhibition in cell line MDA-MB-231; Veh vs STM2457 (E). Genes were considered significant with a fold change (FC) of ≥1.5 or ≤-1.5 and a FDR < 0.05.

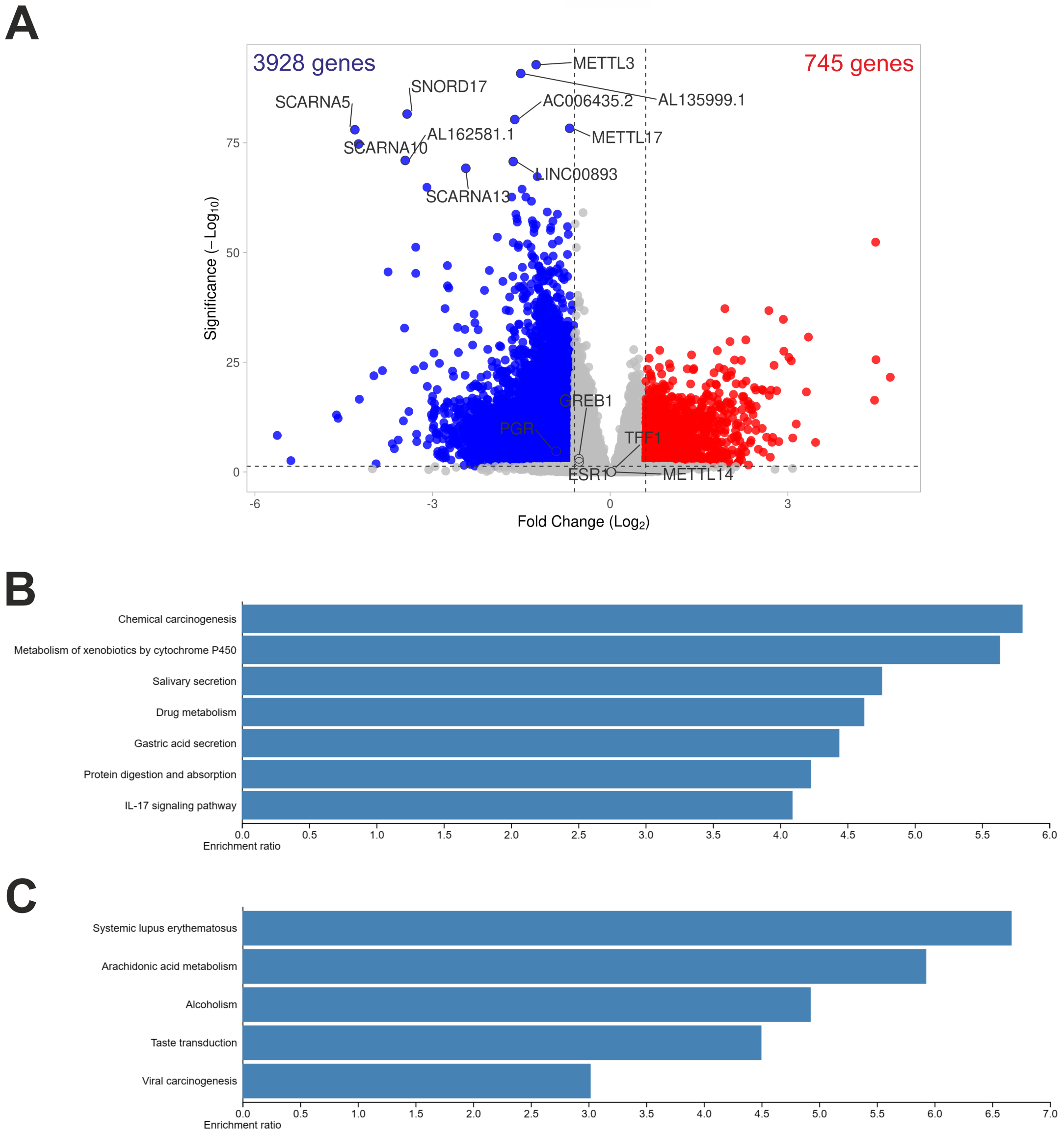

A
3928 genes
745 genes
METTL3
AL135999.1
SNORD17
SCARNA5
AC006435.2
AL162581.1
METTL17
SCARNA10
LINC00893
SCARNA13
GREB1
PGR
TFF1
ESR1
METTL14
Significance (−Log10)
Fold Change (Log2)
-6
-3
0
3
0
25
50
75
B
Chemical carcinogenesis
Metabolism of xenobiotics by cytochrome P450
Salivary secretion
Drug metabolism
Gastric acid secretion
Protein digestion and absorption
IL-17 signaling pathway
0.0
0.5
1.0
1.5
2.0
2.5
3.0
3.5
4.0
4.5
5.0
5.5
6.0
Enrichment ratio
C
Systemic lupus erythematosus
Arachidonic acid metabolism
Alcoholism
Taste transduction
Viral carcinogenesis
0.0
0.5
1.0
1.5
2.0
2.5
3.0
3.5
4.0
4.5
5.0
5.5
6.0
6.5
7.0
Enrichment ratio

Supplementary Figure 20 Comparison of high and low *METTL3* expression in BCa tumour specimens

Differential gene expression between low and high *METTL3* expression in BCa tumour specimens utilising TCGA data (n=1078). (A) Volcano plot displays DEGs between Q1 and Q4 of *METTL3* expression (log2FC of $\geq \pm 1$, -log10 padj >1.3). Enriched KEGG pathways were determined for the significant DEGs that were increased when *METTL3* expression was (B) low (Q1) and (C) high (Q4) (FDR < 0.05).

Supplementary Table 1. Correlations of METTL3, METTL14 and CBLL1 expression with expression of other proteins implicated in breast cancer assessed via IHC in the Nottingham BCa cohort. Statistical p values determined by two-tailed Spearman's correlation and $p \leq 0.05$ highlighted in bold.

Protein		METTL3	METTL14	CBLL1
	Correlation coefficient		**0.748**	**0.353**
**METTL3**	p value		**p <0.0001**	**p <0.0001**
	N		**652**	**539**
	Correlation coefficient	**0.748**		**0.361**
**METTL14**	p value	**p <0.0001**		**p <0.0001**
	N	**652**		**683**
	Correlation coefficient	**0.353**	**0.361**	
**CBLL1**	p value	**p <0.0001**	**p <0.0001**	
	N	**539**	**683**	
	Correlation coefficient	**0.338**	**0.33**	**0.249**
**CMYC (Cytoplasmic)**	p value	**p <0.0001**	**p <0.0001**	**p <0.0001**
	N	**670**	**753**	**616**
	Correlation coefficient	**0.283**	**0.264**	**0.309**
**AR**	p value	**p <0.0001**	**p <0.0001**	**p <0.0001**
	N	**757**	**879**	**687**
	Correlation coefficient	**0.168**	**0.199**	**0.186**
**E-cadherin**	p value	**p <0.0001**	**p <0.0001**	**p <0.0001**
	N	**788**	**910**	**711**
	Correlation coefficient	**0.299**	**0.305**	**0.192**
**ER alpha**	p value	**p <0.0001**	**p <0.0001**	**p <0.0001**
	N	**812**	**944**	**745**
	Correlation coefficient	**0.193**	**0.192**	**0.241**
**FOXA1**	p value	**p <0.0001**	**p <0.0001**	**p <0.0001**
	N	**546**	**627**	**506**
	Correlation coefficient	**0.19**	**0.258**	**0.169**
**BRCA1 (Nuclear)**	p value	**p <0.0001**	**p <0.0001**	**p <0.0001**
	N	**673**	**782**	**616**
	Correlation coefficient	0.022	**0.072**	**-0.081**
**BRCA1 (Cytoplasmic)**	p value	p 0.561	**p 0.044**	**p 0.043**
	N	671	**780**	**615**
	Correlation coefficient	-0.002	-0.059	-0.083
**BRCA2 (Nuclear)**	p value	p 0.959	p 0.143	p 0.062
	N	523	607	503
	Correlation coefficient	**0.186**	**0.198**	**0.142**
**BRCA2 (Cytoplasmic)**	p value	**p <0.0001**	**p <0.0001**	**p 0.001**
	N	**524**	**608**	**504**

Supplementary Table 2. Alternative splicing analysis following RNA-Seq of MCF7 and MDA-MB-231 cells subjected to siRNA-mediated knockdown of METTL3, METTL14 or CBLL1. Differentially spliced transcript and gene numbers are detailed for each alternative splicing event type. (A) Number of significant alternative splicing events compared between MCF7 treated with either siSCR or si*METTL3* and with Veh or E2 (n=3), and between MDA-MB-231 treated with either siSCR or si*METTL3* (n=3). (B) Number of significant alternative splicing events compared between MCF7 cells treated with either siSCR or si*METTL14* and with Veh or E2 (n=3), and between MDA-MB-231 treated with either siSCR or si*METTL14* (n=3). (C) Number of significant alternative splicing events compared between MCF7 cells treated with either siSCR or si*CBLL1* and with Veh or E2 (n=3), and between MDA-MB-231 cells treated with either siSCR or si*CBLL1* (n=3). Significant alternative splice event= dPSI ≥5% and FDR < 0.05. SE = skipped exon; A5SS = alternative 5' splice site; A3SS = alternative 3' splice site; MXE = mutually exclusive exon; RI = retained intron.

A

	MCF7				MDA-MB-231
	si*SCR* Veh vs si*SCR* +E2	si*METTL3* Veh vs si*METTL3* +E2	si*SCR* Veh vs si*METTL3* Veh	si*SCR* +E2 vs si*METTL3* +E2	si*SCR* vs si*METTL3*
SE transcripts	2954	2213	1723	2896	1751
SE genes	1957	1517	1239	1897	1264
A5SS transcripts	605	484	377	736	306
A5SS genes	533	420	338	611	270
A3SS transcripts	622	419	379	602	242
A3SS genes	517	369	325	515	221
MXE transcripts	2702	701	542	3429	600
MXE genes	1620	464	372	1920	414
RI transcripts	514	290	302	623	269
RI genes	457	270	282	533	251

B

	MCF7				MDA-MB-231
	si*SCR* Veh vs si*SCR* +E2	si*METTL14* Veh vs si*METTL14* +E2	si*SCR* Veh vs si*METTL14* Veh	si*SCR* +E2 vs si*METTL14* +E2	si*SCR* vs si*METTL14*
SE transcripts	2222	2459	1218	1457	1486
SE genes	1505	1667	920	1090	1091
A5SS transcripts	488	504	321	350	304
A5SS genes	423	429	279	315	271
A3SS transcripts	387	429	266	358	233
A3SS genes	340	378	246	321	205
MXE transcripts	707	717	430	614	535
MXE genes	455	472	292	398	403
RI transcripts	256	265	170	179	128
RI genes	246	252	167	170	126

C

	MCF7				MDA-MB-231
	si*SCR* Veh vs si*SCR* +E2	si*CBLL1* Veh vs si*CBLL1* +E2	si*SCR* Veh vs si*CBLL1* Veh	si*SCR* +E2 vs si*CBLL1* +E2	si*SCR* vs si*CBLL1*
SE transcripts	2213	3090	1199	1435	2822
SE genes	1467	1850	913	1044	1871
A5SS transcripts	479	507	238	280	577
A5SS genes	414	427	218	250	495
A3SS transcripts	323	470	276	271	489
A3SS genes	285	397	246	237	421
MXE transcripts	914	1033	537	555	761
MXE genes	546	640	370	386	512
RI transcripts	256	386	238	126	820
RI genes	236	344	218	123	694

Supplementary Table 3. Alternative splicing analysis following STM2457 treatment of MCF7 and MDA-MB-231. Differentially spliced transcript and gene numbers are detailed for each alternative splicing event type. (A) Number of significant alternative splicing events compared between MCF7 cells treated with either Vehicle or STM2457 and with Vehicle or E2 (n=3), and between MDA-MB-231 cells treated with either Vehicle or STM2457 (n=3). Significant alternative splice event= dPSI ≥5% and FDR < 0.05. SE = skipped exon; A5SS = alternative 5' splice site; A3SS = alternative 3' splice site; MXE = mutually exclusive exon; RI = retained intron.

A

	MCF7				MDA-MB-231
	Veh vs +E2	STM2457 Veh vs STM2457 +E2	Veh vs STM2457 Veh	+E2 vs STM2457 +E2	Veh vs STM2457
SE transcripts	3797	5854	3477	5054	2858
SE genes	2249	3033	2055	2748	1718
A5SS transcripts	696	920	714	865	466
A5SS genes	595	751	592	684	421
A3SS transcripts	694	968	722	844	422
A3SS genes	555	754	573	649	373
MXE transcripts	1258	1704	1168	1653	844
MXE genes	734	938	625	853	527
RI transcripts	700	1017	913	1034	459
RI genes	573	825	744	793	405
